# Biochemical and Biophysical Divergences between Two l‑Asparaginase II Variants: Potential for Using EcA2-K12 as a Biosimilar

**DOI:** 10.1021/acs.biochem.4c00663

**Published:** 2025-04-16

**Authors:** Talita Stelling de Araujo, Anna Catharinna da Costa, Camila Dias Leite da Silva, Fernando de Sá Ribeiro, Rafael Alves de Andrade, Heitor Affonso Paula Neto, Renato Sampaio Carvalho, Luís Maurício T. R. Lima, Marcius da Silva Almeida

**Affiliations:** † Protein Advanced Biochemistry (PAB), Institute of Medical Biochemistry (IBqM)-National Center for Structural Biology and Bioimaging (CENABIO), 28125 Universidade Federal do Rio de Janeiro, Rio de Janeiro, Rio de Janeiro 21941-902, Brazil; ‡ Laboratório de Biotecnologia Farmacêutica (pbiotech), Faculdade de Farmácia, Universidade Federal do Rio de Janeiro, Rio de Janeiro, Rio de Janeiro 21941-902, Brazil; § Programa de Pós-Graduação em Química Biológica, Universidade Federal do Rio de Janeiro, Rio de Janeiro, Rio de Janeiro 21941-902, Brazil; ∥ Laboratório de Alvos Moleculares (LAM), Faculdade de Farmácia, Universidade Federal do Rio de Janeiro, Rio de Janeiro, Rio de Janeiro 21941-902, Brazil; ⊥ Programa de Pós-graduação em Imunologia e Inflamação, Universidade Federal do Rio de Janeiro, Rio de Janeiro, Rio de Janeiro 21941-902, Brazil

## Abstract

l-asparaginase II (EcA2) is essential for treating Acute Lymphoblastic Leukemia, the most common childhood cancer. This enzyme catalyzes the hydrolysis of l-asparagine or l-glutamine to ammonia and l-aspartate or l-glutamate. The first FDA-approved EcA2 biopharmaceutical, Elspar, was introduced in 1978, followed by other biosimilars. Despite stringent approval criteria, variations in plasmatic activity and therapeutic efficacy persist across different EcA2 preparations, often leading to substandard product notifications. Many studies focus on the EcA2 from the K12 strain (EcA2-K12), which differs by four amino acids from reference biopharmaceuticals, including Elspar (EcA2-4M). Here, we show that EcA2-4 M has over twice the specific activity on both the hydrolysis of l-asparagine and on human lymphoblast cells compared to EcA2-K12. EcA2-K12 demonstrates 4-fold greater specificity for l-asparagine over l-glutamine, considering their *k*_cat_, but similar *K*_M_ toward each amino acid. Interestingly, EcA2-K12 has 3-fold lower affinity for l-aspartate, linked to reduced stabilization of its N-terminal active site loop. Although both variants exhibit indistinguishable thermostability, EcA-K12 shows a higher tendency to oligomerize. We solved the 3D structures of both variants by X-ray crystallography, and normal-mode analysis revealed wider conformational changes in EcAK12’s active site. Our data indicate that EcA2-K12 has lower activity due to the higher conformational dynamics of the N-terminal active site loop. Nevertheless, EcA2-K12 is a beneficial alternative or complement to existing therapeutic schemes with EcA2-4M, due to its higher specificity to l-asparagine, which is of fundamental importance since activity on l-glutamine is associated with harmful side effects.

## Graphical abstract

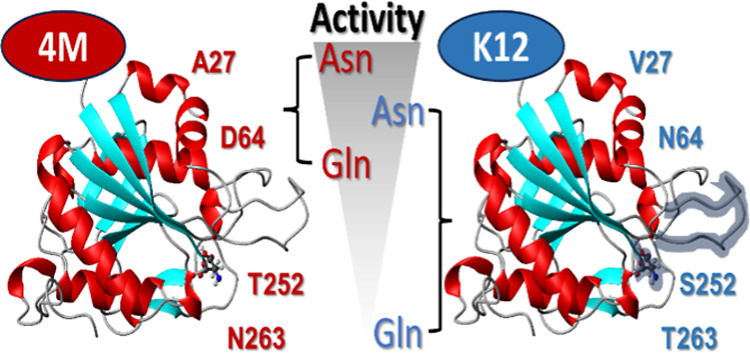

## Introduction

l-asparaginase II (EC 3.5.1.1) is an amidohydrolase that primarily catalyzes the hydrolysis of l-Asn to l-Asp and ammonia, but that can also act on other substrates, including l-Gln.^1^ This enzyme has been used for over 40 years in the treatment of leukemias, particularly Acute Lymphoblastic Leukemia (ALL), which is the most prevalent cancer in childhood.^2^ The therapeutic use of this enzyme leads to a cumulative survival rate of at least about 70% of patients with ALL after 5 years from the end of treatment.^3^ The l-asparaginase II (EcA2) and its variants, including the long-acting polyethylene glycol-conjugated version, pegaspargase,^4,5^ are considered essential medicines by the World Health Organization (WHO Model Lists of Essential Medicines, ATC code L01XX02).^1,6^ Since its isolation,^6–8^ research groups have investigated the structural, biochemical and pharmacological properties of l-asparaginase II. These studies have provided a comprehensive description of this biopharmaceutical, regarding its catalytic mechanism, stability, pharmacokinetics and adverse effects, and have been extensively reviewed.^9–15^ Much of the current research effort on l-asparaginase II is focused on the development of variants with lower toxicity, higher plasma half-life, and improved storage stability.^16–18^

The first biopharmaceutical with l-asparaginase II from available for ALL treatment was Elspar, approved by the FDA in 1978. Since then, a large set of products has been made available by other manufacturers. Biosimilars, although not identical to their originator product, are expected to be highly similar to the licensed biologic product, with their molecular integrity, physicochemical identity, and clinical safety and efficacy demonstrated by comparability studies. Differences between the proposed biosimilar and the reference product must be supported by strong scientific evidence that these differences are not clinically meaningful.^19,20^

Despite the stringent requirements for biosimilar approval, discrepancies in plasmatic activity between currently available asparaginase preparations are continually reported.^21–25^ Motivated by these reports, we conducted a comparability study between two of these EcA2 preparations, Aginasa (Medac, GmbH) and Leuginase (Beijing SL Pharmaceutical, China), which are reported to exhibit divergent plasmatic activity and therapeutic efficacy, despite showing comparable in vitro enzymatic activity.^26^ Extensive biophysical characterization allowed the identification of differences in formulation, conformation, oligomeric distribution, and molecular mass of the two biopharmaceuticals analyzed. These differences could explain the distinct bioavailability observed, and diverse clinical outcomes.^22^

At least two types of EcA2 with different amino acid sequences can be found in l-asparaginase biosimilars, both of which differ from the enzyme sequence of the K12 strain. The EcA2-K12 is the enzyme used in the vast majority of studies on l-asparaginases, as it is the reference for amino acid sequence of the enzyme produced by , available in UniProt (accession code: P00805). The most prevalent amino acid sequence observed in EcA2 biopharmaceuticals, including pegylated forms such as Pegaspargase and Calaspargase, as well as the reference product Elspar, differs from that of the K12 strain by four amino acid substitutions—V27A, N64D, S252T, and T263N—and is referred to here as EcA2-4M.^10^ This EcA2-4M sequence corresponds to asparaginase from other strains, including BL21­(DE3) (GenBank: CAQ33267; ACN38272.1).

Based on these findings, we produced two of the most cited variants of l-asparaginase, EcA2-K12 and EcA2-4M, to evaluate their conformation, activity, and specificity to their substrates. We demonstrated that the differences their amino acid sequences lead to variations in enzymatic activity, substrate specificity, effects on a leukemic cell line, protein dynamics, and conformational stability in solution, despite their nearly identical 3D crystal structures.

## Results

### Production of EcA2-K12 and EcA2-4M

Two EcA2 variants differing in four amino acids belong to two commonly used strains (Figure 1): the K12 (Uniprot: P00805), and the BL21­(DE3) strain. The EcA2 from BL21­(DE3), hereby cited as EcA2-4M, is the prevalent form used in commercial pharmaceutical preparations of l-asparaginase, including Elspar (the innovator product, approved by the FDA in 1978), Spectrila, and their PEGylated versions, respectively Oncaspar and Asparlas.

**Figure 1:**
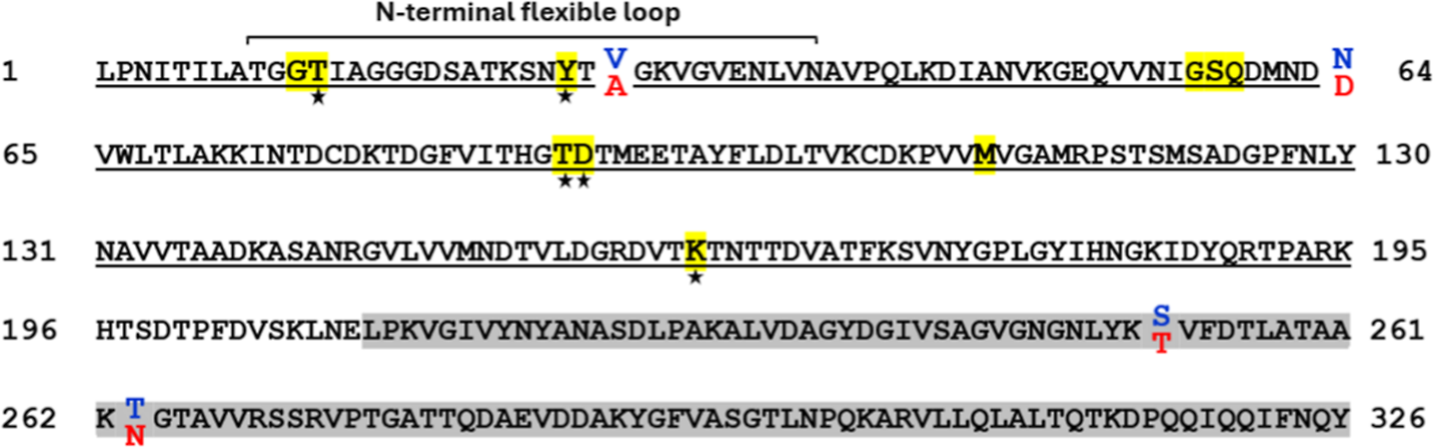
Amino acid sequences of EcA2-K12 and EcA2-4M. The four amino acid differences between the variants EcA2-K12 and EcA2-4 M are shown in blue and red, respectively. The flexible N-terminal loop boundaries of EcA2 are indicated in the figure. Residues that form the catalytic site are highlighted with yellow boxes, with those directly involved in catalysis indicated by stars. The N-terminal domain amino acids are underlined, while the C-terminal domain is shaded in gray.

To compare these two EcA2 variants we expressed and purified both using the same workflow as depicted in the Materials and Methods section. Both proteins were secreted and purified from the medium by diafiltration, followed by hydrophobic interaction chromatography, and finally by anion exchange chromatography. This purification procedure resulted in approximately 102 mg of EcA2 per liter of culture, with purity over 97%, as determined by analyzing pixel intensity histograms generated using ImageJ software, from SDS-PAGE of the purified samples (Figure S1).^27^ The integrity of the two asparaginase products was confirmed by mass spectrometry (Figure S1). Both EcA2 have an average molecular mass of 34,591.94 Da and a monoisotopic molecular mass of 34,570.56 Da, considering the presence of one disulfide bond, as calculated with the Compute pI/Mw tool.^28^

### Enzymatic Activity and Kinetics Parameters

The specific activity of EcA2-K12 and EcA2-4 M was assessed by monitoring the hydrolysis of the l-Asn in vitro (Figures 2A and S2). These results demonstrate that the EcA2-4 M variant exhibits approximately twice the specific activity of EcA2-K12, with values of 69 ± 4 and 25 ± 2 IU/mg, respectively. Cytotoxic activity of EcA2 variants was evaluated against acute lymphoblastic leukemia cell line CCRF-CEM (Figures 2B and S3), resulting in higher activity for EcA2-4M, with IC_50_ values of 4.1 ± 0.5 nM compared to 9.0 ± 2.1 nM for EcA2-K12, lying in the same order of magnitude.

**Figure 2:**
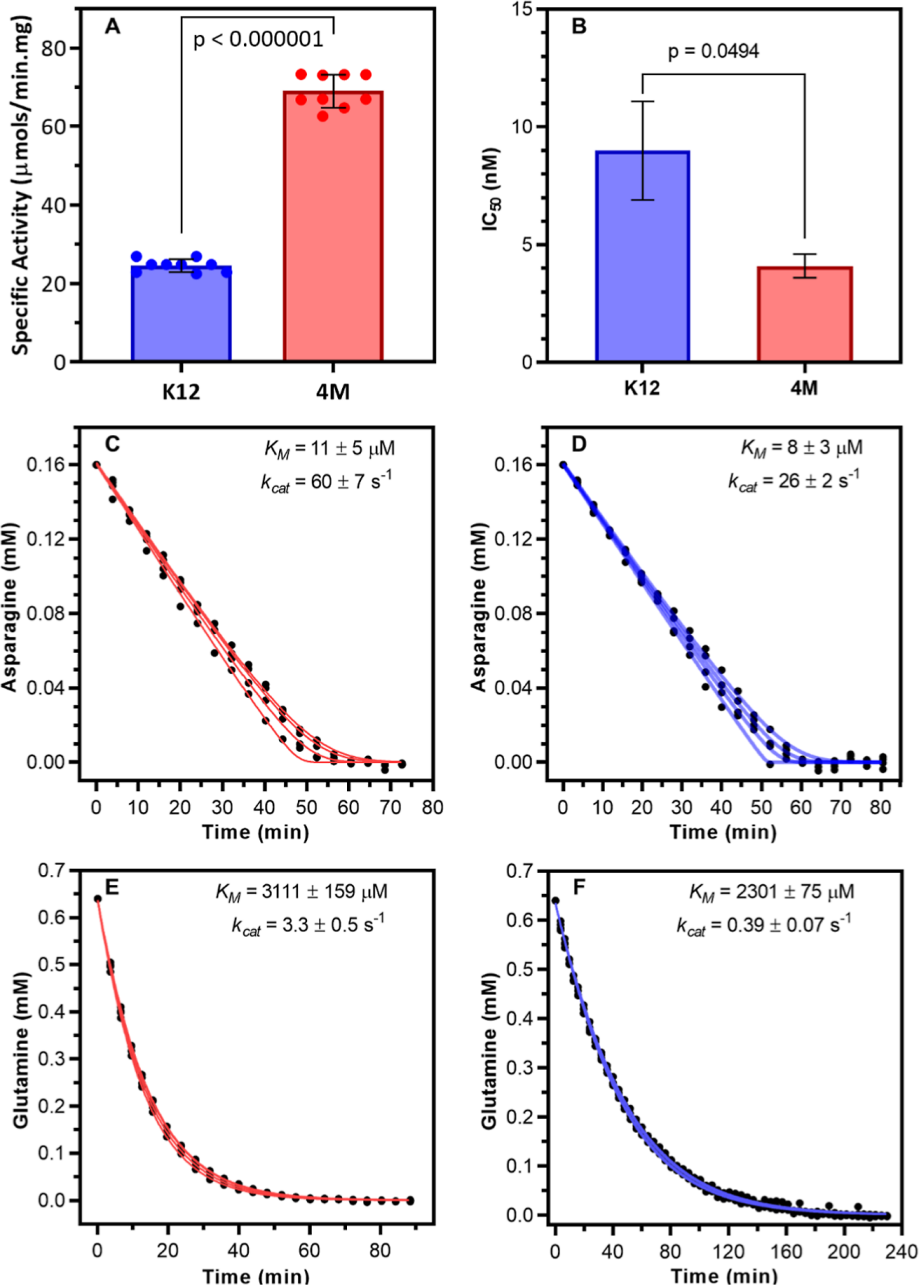
Activity of EcA2-4 M is significantly higher than EcA2-K12. (A) The specific activity of l-Asn hydrolysis was measured with l-asparaginase at 25 °C and pH 8.0. Bars represent the mean, and the error bars indicate the standard deviations. Statistical significance determined using the Holm–Sidak method, with alpha = 0.05 (*n* = 9). (B) The effect of l-asparaginases on cells. The experimental data were fit to a dose response equation with variable slope, shown in Figure S3 (*n* = 3, *R* ^2^ = 0.92 and 0.97 for EcA2-K12 and EcA2-4M, respectively) Bars represent the mean, and the error bars indicate the standard deviations. (C,D) Kinetics of 160 μM l-Asn hydrolysis measured by NMR at 25 °C and pH 8.0 (*n* = 4). (E,F) Kinetics of 640 μM l-Gln hydrolysis measured by NMR at 25 °C and pH 8.0 (*n* = 4). Standard deviations were calculated from independent fits of four experimental data sets.

The hydrolysis reaction catalyzed by EcA2 variants was monitored by 1D (^1^H)-NMR using the substrates l-Asn or l-Gln. Integration of the H_α_ peaks of l-Asn or l-Asp and the H_γ_ peaks of l-Gln or l-Glu over time (Figure S4) was used to calculate substrate consumption (Table S1 and Figure 2C,F). The *K*_M_ values obtained for the variants were similar, indicating comparable apparent affinity. However, the results show that the EcA2-4 M variant exhibits a substrate turnover rate (*k*_cat_) approximately twice that of EcA2-K12, which can explain the differences in specific activity and cytotoxicity.

Similarly, for l-Gln, comparable *K*_M_ values were observed for both EcA2-4M and EcA2-K12, but the substrate turnover rate of EcA2-4M was approximately 10 times higher than that of EcA2-K12. These findings suggest that, despite EcA2-K12 displaying lower catalytic activity, it is more specific toward l-Asn.

### Interaction of EcA2-K12 and EcA2-4M with l-Asp

We evaluated the binding affinity of the EcA2 variants by monitoring variations in its spectral center of mass of intrinsic tryptophan fluorescence emission in the presence of increasing concentrations of l-Asp (Figures S5 and S6). The analysis of the binding curves obtained from this experiment allowed for the calculation of the dissociation constant (*K*_D_) of the enzymes for l-Asp (Figure 3). This analysis demonstrated that the EcA2-K12 variant has a dissociation constant for the product of the catalyzed reaction that is three times higher than that of the EcA2-4M variant, with *K*_D_ values of 211 ± 52 and 66 ± 14 μM, respectively.

**Figure 3:**
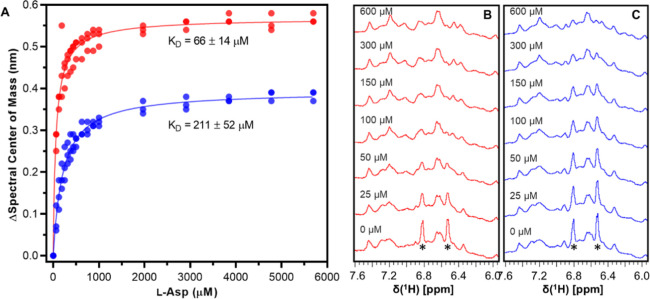
Affinity for l-Asp is higher for EcA2-4M than EcA2-K12. (A) Binding of l-Asp to EcA2-4M (red, *n* = 3) or EcA2-K12 (blue, *n* = 3) determined by intrinsic tryptophan fluorescence. Standard deviations for the mean *K*_D_ values were calculated from independent fits of three experimental data sets for each EcA2 variant. (B,C) Multiple displays of NMR-titration analysis of EcA2-4M (red) or EcA2-K12 (blue) at 160 μM supplemented with increasing concentrations of l-Asp. Concentration of l-Asp used for the acquisition of each spectrum is indicated in the figure. For each experiment, a total of 256 scans with 2 s of relaxation delay were collected in a 600 MHz spectrometer at 298 K. Asterisks indicate the peaks of two aromatic protons previously attributed to Y25.

Additionally, we assess the ability of the ligand l-Asp to induce a conformational response in the enzymes using titration analyses, monitoring the two sharp aromatic resonances previously attributed to the aromatic protons of Tyr25 in the EcA2 variants by 1D (^1^H)-NMR (Figure 3).^29^ The titration with l-Asp indicates that the EcA2 4 M variant requires a lower concentration of ligand, 100–150 μM, for the stabilization of its closed form, evidenced by the disappearance of the H^ε^ and H^δ^ signals from the aromatic ring of Tyr25. However, for the EcA2-K12 variant, the disappearance of the Tyr25 resonances is only observed in the presence of >600 μM of l-Asp. This observation confirms that the EcA2-4M variant has a higher affinity for l-Asp and that the binding of this amino acid to the catalytic site is accompanied by the closure of the N-terminal loop.

Saturation transfer difference (STD-NMR) experiments were conducted to determine the binding epitope l-Asp to the enzymes (Figure 4). The relative saturation percentages of each ligand proton were estimated using the calculated values of amplification factors (A_STD_), which quantitatively represents the efficiency of the saturation transfer from the protein to the proton and consequently reflects the proximity of these protons to the protein.^30^ The saturation efficiency for both variants was remarkably higher for the H^α^ of l-Asp than for H^β2^ and H^β3^, in a similar proportion, indicating that H^α^ is closer to the protein. This result demonstrates that the binding modes of l-Asp to the active site of both EcA2 variants are not discernible.

**Figure 4:**
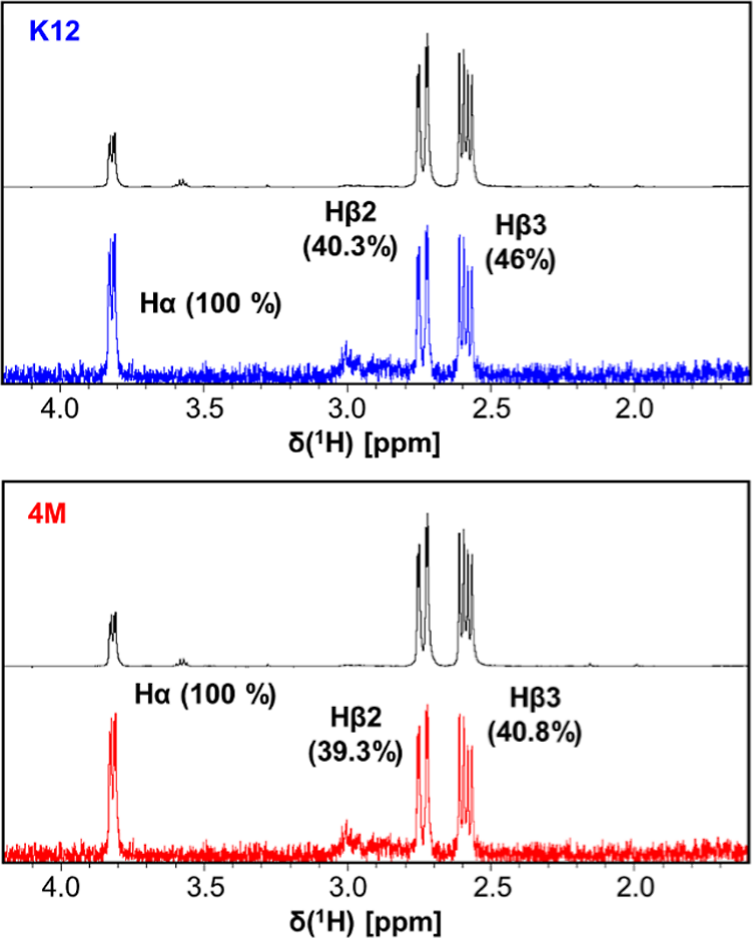
STD-NMR analyses reveal that binding epitope of l-Asp to EcA2-4M e EcA2-K12 is very similar. The reference spectrum acquired for each ligand is in black, and the corresponding STD-NMR spectrum is colored in blue (EcA2-K12) or red (EcA2-4M). The assigned protons are indicated in each spectrum with the respective relative STD percentages in parentheses.

### Comparative Conformational Analysis of EcA2-K12 and EcA2-4M Reveals Very Similar but Not Identical Structures in Solution

A molecular characterization of EcA2 variants was conducted in an attempt to identify the molecular basis underlying the observed catalytic differences. First, we assessed the oligomeric distribution of EcA2 variants using analytical size-exclusion chromatography (Figure 5). The resulting chromatograms indicated that EcA2-4M and EcA2-K12 display a similar oligomeric distribution, with a major peak corresponding to the EcA2 tetramer,^26^ preceded by smaller peaks associated with the presence of higher hydrodynamic radii species of EcA2 in the protein preparations. Despite these similarities, the chromatogram obtained for the EcA2-4M shows a smaller contribution of larger hydrodynamic radii species, with a prepeak percentage of the total area of approximately 5% compared to 13% for EcA2-K12. Additionally, the main peak for the EcA2-4M elutes with a significantly shorter retention time, indicating species with higher hydrodynamic radii or with weaker association with the chromatographic matrix. These findings may reflect a lower aggregation propensity for EcA2-4M but with the main oligomeric specie being significantly wider than EcA2-K12.

**Figure 5:**
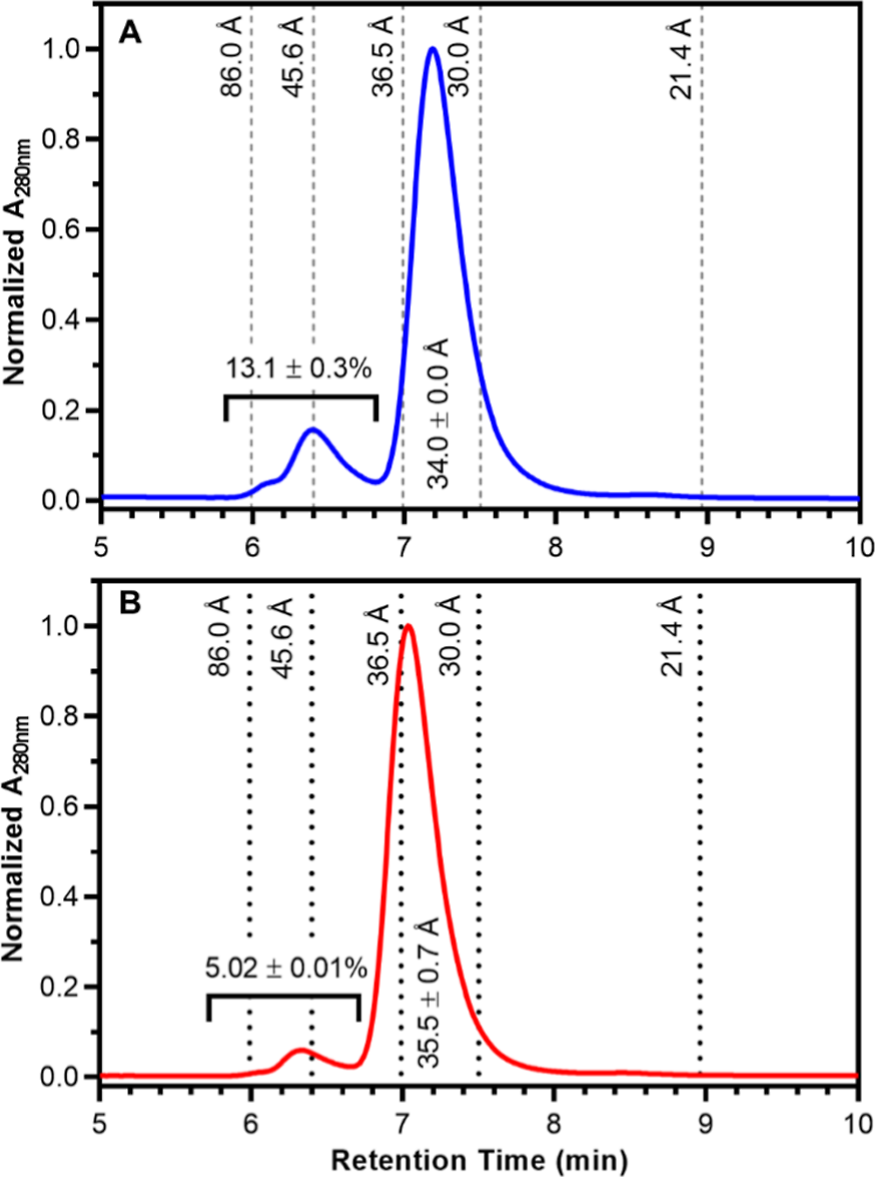
EcA2-4M exhibits a distinct oligomeric distribution compared to EcA2-K12. Analytical size-exclusion chromatography was performed on EcA2-K12 (A) and EcA2-4M (B). The elution profiles of protein standards are indicated by dashed lines, along with their respective hydrodynamic radii. The standard curve correlating the retention time with the hydrodynamic radii of the standards is shown in Figure S7. The hydrodynamic radius (Å) of the main peak the estimated abundance (% of total peak area) of high molecular weight species (pré-peaks), and their standard deviations from three independent measurements are indicated in the graph.

The secondary structure content of the EcA2 variants is similar, as judged by the similar circular dichroism spectra, with indication of a protein mainly with well-defined α-helices and β-strands, owing to a large negative band at 209–223 nm and a positive band bellow 200 nm (Figure 6A). Their intrinsic fluorescence spectra (Figure 6B) are also equivalent, both with a typical pattern of buried tryptophan residue not exposed to aqueous milieu. The 1D [^1^H]-NMR spectra of each EcA2 variant are also equivalent, with wide amide and aromatic proton signal dispersion typical of globular proteins (Figure 6C). Minor differences in the obtained spectra (highlighted with gray shades in Figure S8) might reflect the differences in the amino acid composition of the produced enzymes and can therefore be used as spectral signatures for identifying the EcA2 variants.

**Figure 6:**
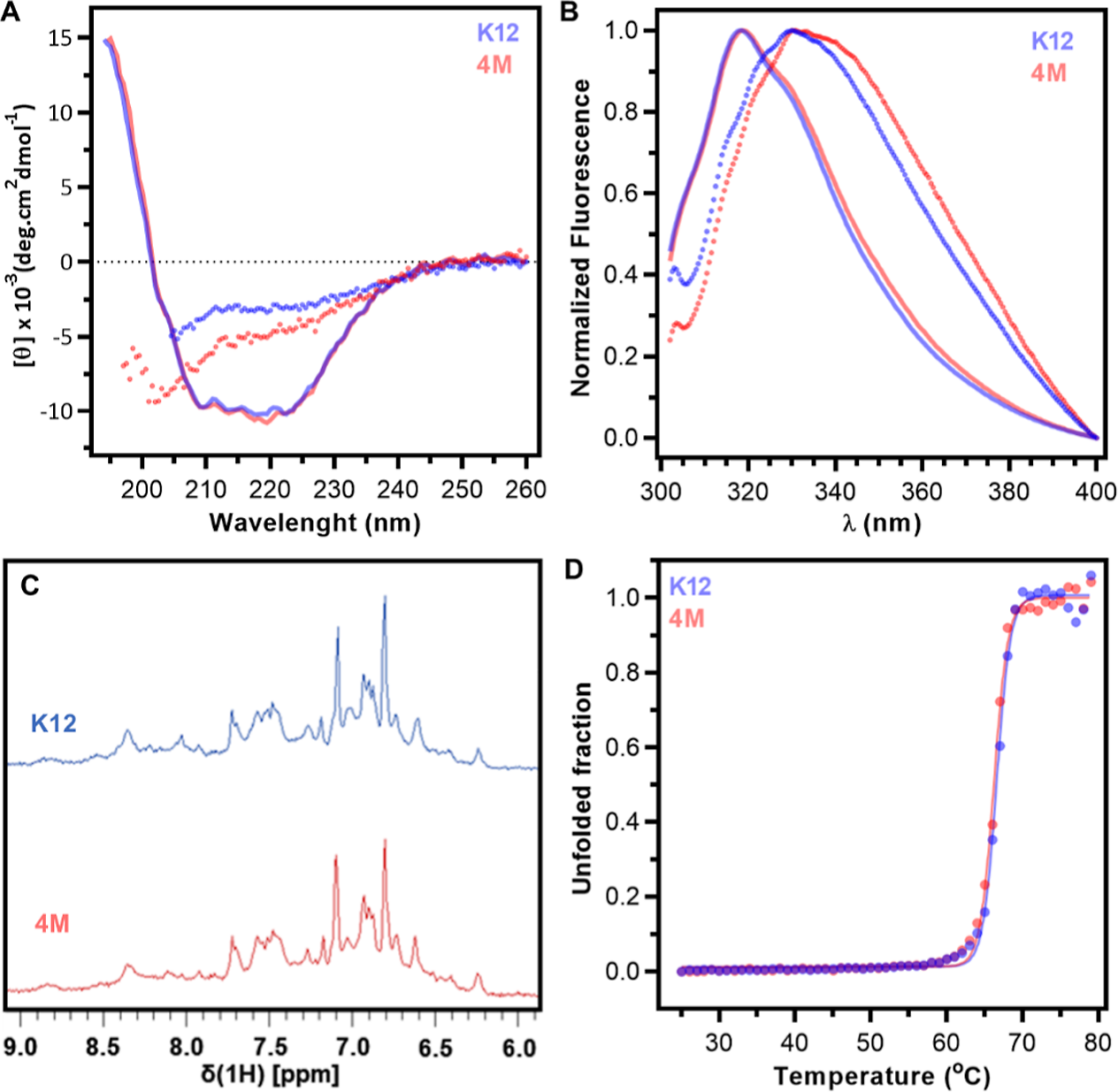
Solution conformation and thermal stability of EcA2-K12 and EcA2-4M are similar. (A) Circular dichroism spectra recorded with proteins at 25 °C (solid lines) or 80 °C (dots). (B) Normalized intrinsic fluorescence spectra recorded at 25 °C (solid lines) or 70 °C (dots). (C) Amide and aromatic region of 1D [^1^H]-NMR spectra. NMR spectra were collected at 25 °C and 900 MHz. (D) The unfolded fraction of EcA2 variants at different temperatures was determined by measuring intrinsic fluorescence intensities at 348 nm in normalized spectra (Figure S6). Experimental data were fitted to a two-state model equation with baseline correction (*R* ^2^ > 0.99).

These spectra properties indicate that variations in the primary sequence of the enzymes do not strongly affect their secondary or tertiary structure content in solution. On the other hand, the spectra obtained with the denatured proteins (dotted lines in Figure 6A,B) are significantly distinct. Thermal denaturation experiments monitored by intrinsic fluorescence were performed to evaluate the conformational stability of EcA2 variants (Figures 6D and S9). The melting curves obtained show the presence of two well-defined conformational states, pre- and postabrupt transition, corresponding to the folded and unfolded states of the enzymes, respectively. The melting temperatures are 66.65 ± 0.05 °C for EcA2-K12 and 66.25 ± 0.05 °C for EcA2-4M and indicate that sequence alterations do not affect the solution conformational stability of the enzymes.

### Multiple Monomeric Conformers of EcA2 Variants in Solution by Ion Mobility

The conformational distribution of EcA2 variants was evaluated by ESI-TWIM-MS (Figure 7). The intact average mass of EcA2-K12 and EcA2-4M obtained from the deconvoluted spectra of the ESI-MS is 34,590.00 Da for both proteins, matching the expected mass calculated from the clone sequence (with one cystine), and in agreement with previous results from our group with the Elspar and Aginasa brands corresponding to the EcA2-4M sequence. Ion mobility measurements revealed that both variants similarly populate multiple conformers with dissimilar cross sections. Both EcA2 variants presented two (Figure 7C,G) and even three (Figure 7D,H) well-separated conformers in the traveling-wave domain. Nevertheless, these data indicate that the four-amino acid mutation did not affect the inherent ability of the EcA2 monomers to populate multiple well-defined conformational spaces.

**Figure 7:**
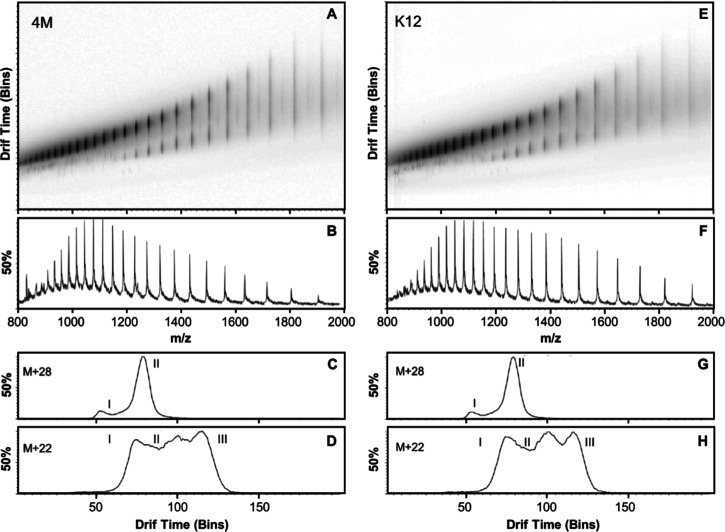
Conformational plasticity evaluation by ion mobility spectrometry–mass spectrometry. ESI-IMS-MS reveals multiple l-asparaginase conformers. EcA2-K12 (A–D) and EcA2-4M (E–H) were evaluated at 5 μM in 0.1% formic acid in water/acetonitrile (50:50). Multiple monomeric conformational states were populated, such as two (C,G) or three (D,H) conformers in the same sample. The deconvoluted spectra revealed a mass of 34,590.0 Da for both EcA2-K12 and EcA2-4M, in agreement with previous data from Aginasa (similar as Elspar) and as expected from the cloned genes.

### Crystallographic Structure of the EcA2 Variants

To understand the structural implications of the four-amino acid differences between EcA2-K12 and EcA2-4M, we solved their crystal structure in the presence of l-Asp under the same crystallization condition and data collection method. All monomers in the asymmetric unit showed the l-Asp in the active site. The monomeric units within each their asymmetric units were similar between the structures, with overall RMSD values lower than 1.5 Å (Figure S10). The monomers of EcA2-K12 and EcA2-4M were essentially superposable, with C^α^ RMSD values lower than 0.4 Å (Figure 8).

**Figure 8:**
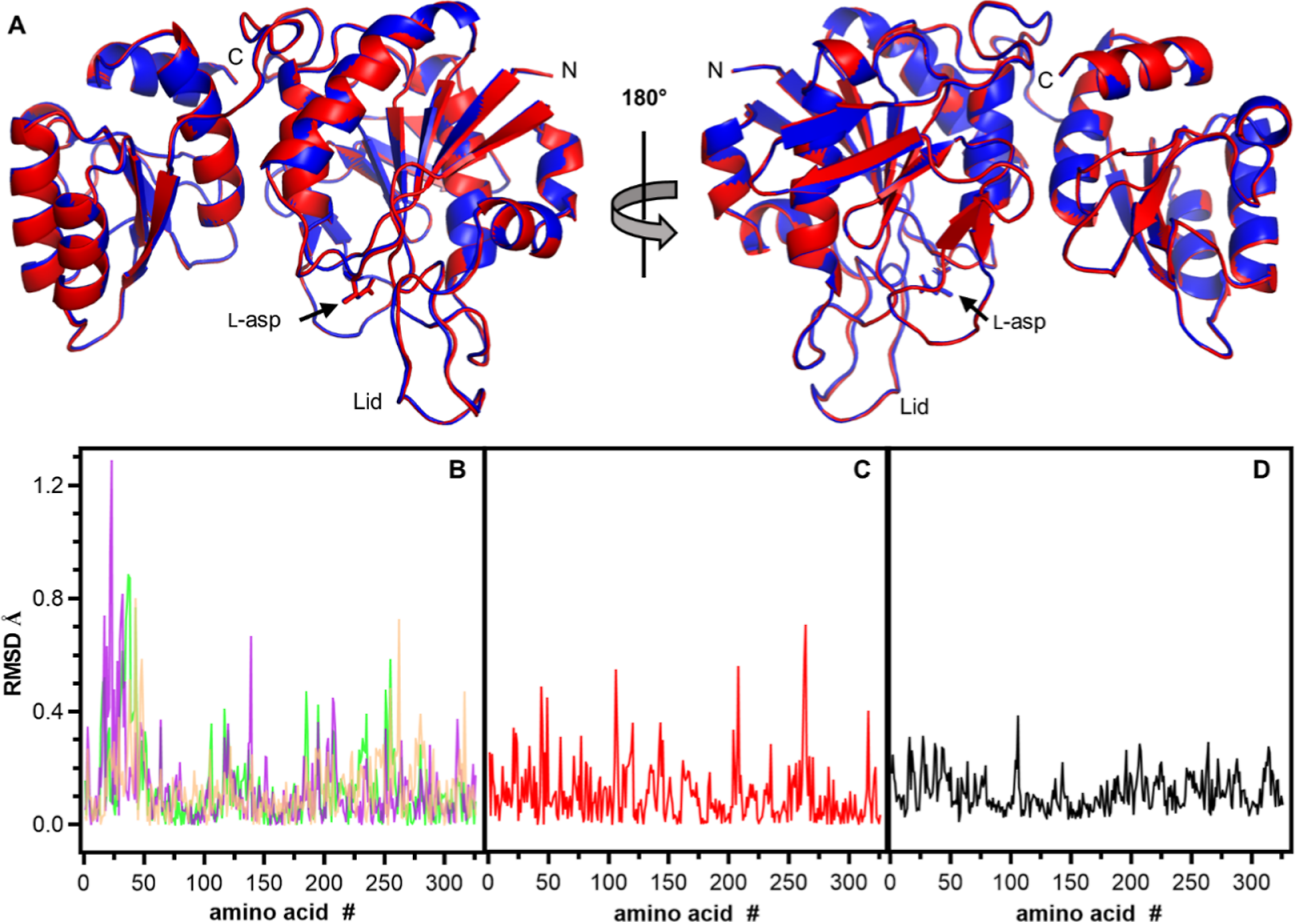
Comparison of crystal structures of EcA2-K12 and EcA2-4M. (A) The chain A of EcA2-K12 (blue) is superimposed with chain A of EcA2-4M (red). The amino acid chain termini are indicated, as well as the loop (lid) that covers the active site and the l-Asp bound to it. C^α^ RMSD of chain alignment by amino acid residue number of (B) EcA2-K12 chains, A vs B (orange); A vs C (magenta); A vs D (green). (C) EcA2-4M chains, A vs B (red). (D) EcA2-4M chain A vs EcA2-K12 chain A (black).

The structural and comparative analysis of EcA2-4M and EcA2-K12 showed a good correspondence between the positions of nonmatching residues and their electron density maps, with the only exception being the valine at position 27 of EcA2-K12 (Figure S11). There was poor correspondence between the electron density map and the spatial position of valine 27, which may be due to the characteristics of the added residue and its location in EcA2-K12. Given its hydrophobicity and constant interaction with a highly solvated region, vital for the opening and closing mechanism of the active site loop, this may lead to greater dynamics compared to alanine 27 of EcA2-4M. These increased dynamics could result in a loss of symmetry for this residue in a crystalline network, leading to the formation of numerous torsional conformations that may hinder or reduce diffraction in the mentioned region. Additionally, it is important to note that the observed differences may be due to inherent crystal disorders originating during the formation of the crystal lattice.

The structures were superposed to highlight the possible differences between the mutated residues (Figures 9 and S12). The findings show good superposition between the reference structure 3ECA and EcA2-4M, indicating clear structural correspondence (Figure S12a). In contrast, the comparison between the reference structure and EcA2-K12 revealed small torsional and positional differences, which are expected due to the different residue side chains (Figure S12b). However, this type of analysis alone does not allow for inferences about changes in dynamics and solution conformational plasticity.

**Figure 9:**
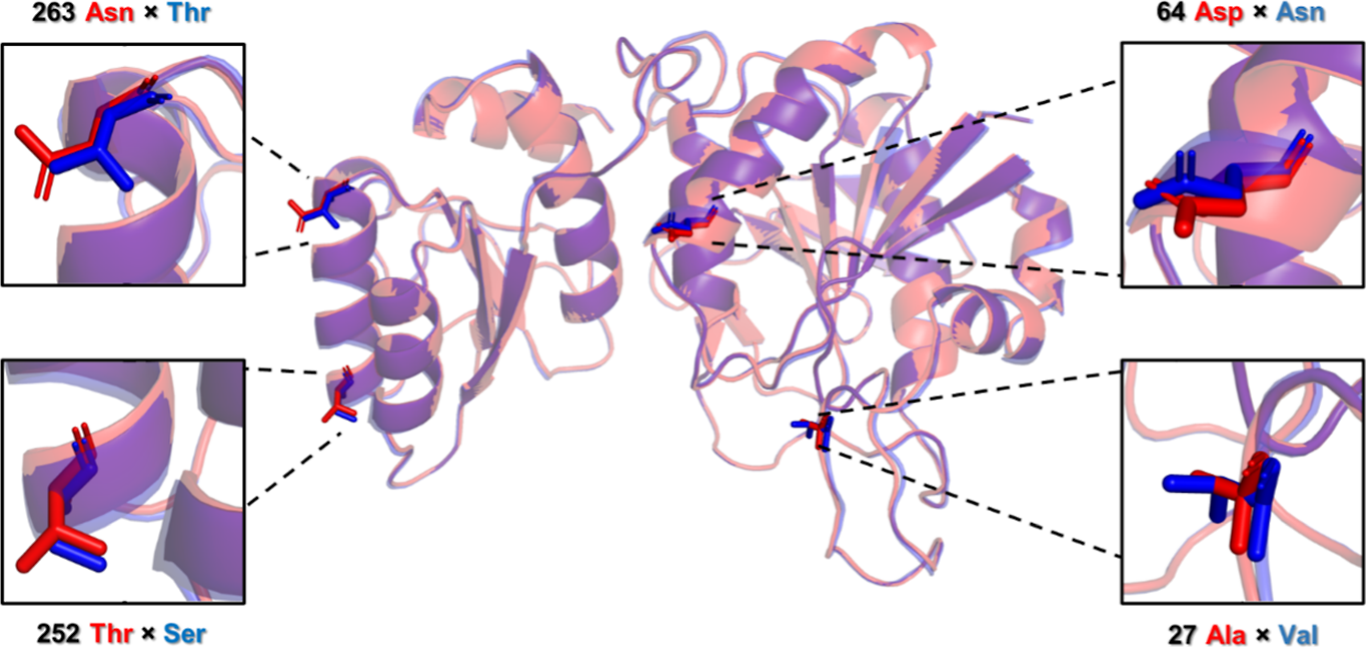
Comparison of the conformation of the nonmatching residues of EcA2-K12 and EcA2-4M. EcA2-4M (red) and EcA2-K12 (blue) are at the same orientation to compare the structures near the nonmatching residues.

### Conformational Plasticity of EcA2 by Normal-Mode Analysis

Since crystal structures cannot precisely predict dynamic behavior in solution, we performed a normal-mode analysis on the obtained 3D crystal structures. This analysis revealed a conformational transition between two distinct states in the tetrameric form, as the technique describes the accessible flexible states of the protein from an equilibrium position. The results indicate two similar, but not identical, chain movement profiles, characterized by ″opening″ and ″closing″ motions (Figure 10). However, when analyzing the trajectory of each C^α^ separately, it is evident that the movement amplitudes are distinct for each chain of each protein (Figure 11). These differences may stem from the mutated residues or the arrangement of the crystal lattice.

**Figure 10:**
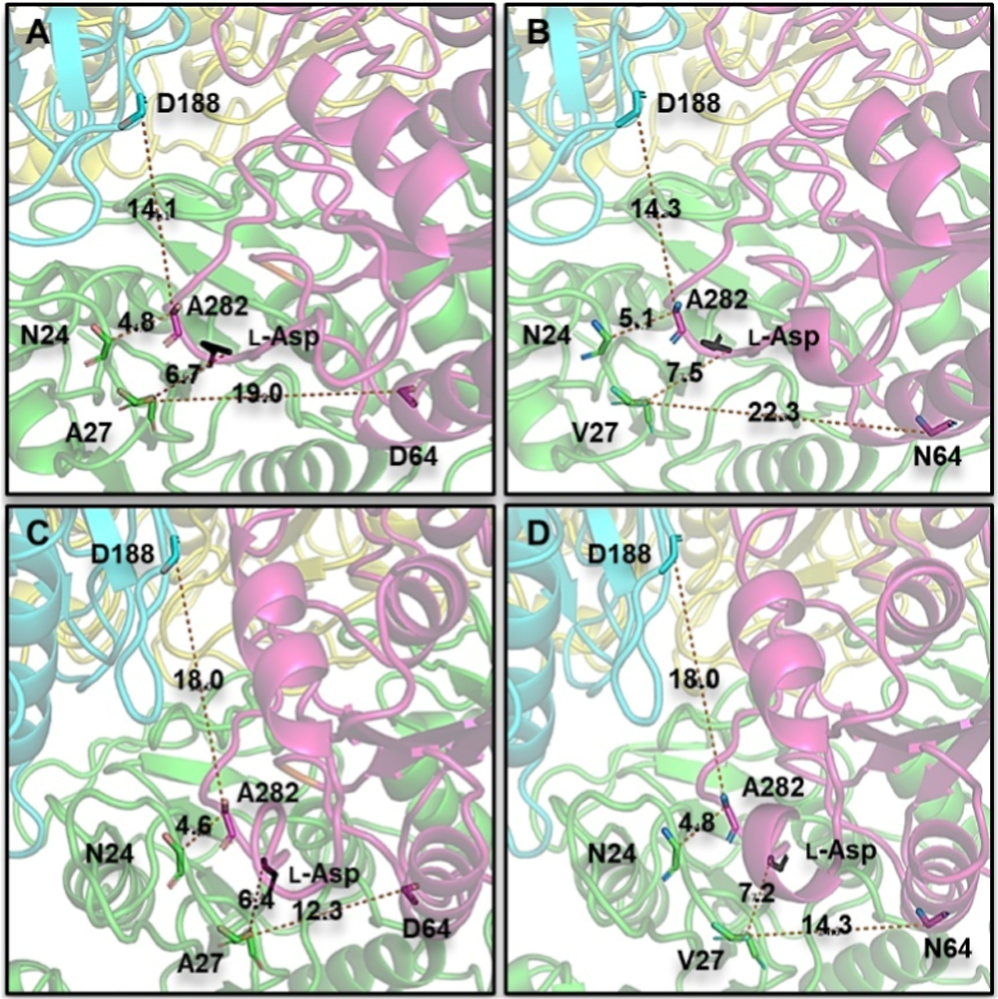
Closeup of the active site showing the open and closed conformations calculated by the normal-mode analysis. (A) EcA2-4M open state. (B) EcA2-K12 open state (C) EcA2-4M close state. (D) EcA2-K12 close state. Each chain is identified by different colors (Green—chain A; Cyan—chain B; Purple—chain C; Yellow—chain D). The distances (Å) to selected amino acids are indicated.

**Figure 11:**
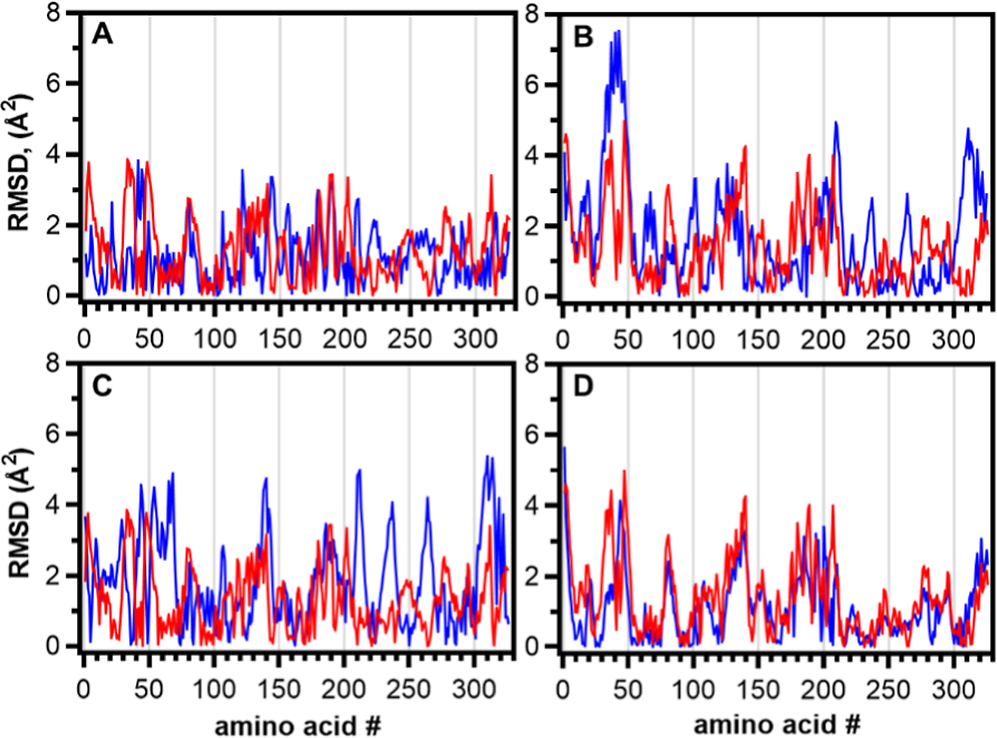
RMSD taken from the normal-mode trajectory file plotted by amino acid number. The graphs present the difference in Å between the structure in equilibrium and the final state, for EcA2-4M (red) or EcA2-K12 (blue). (A) Chain A. (B) Chain B (C) Chain C. (D) Chain D.

## Discussion

Asparaginase from (EcA2) came into focus in the late 1960s after being identified as an effective antitumor enzyme.^31^ Due to its high therapeutic efficacy and cost-effective production methodology, EcA2 remains central to the treatment of acute lymphoblastic leukemia.^32^ Since its therapeutic efficacy peaks at approximately 90% and because it can cause life-threating side effects, ongoing research is mostly focused on developing variants of EcA2.^16,33^ In this work, we systematically evaluate the kinetic parameters and structural properties of two variants of the l-asparaginase II enzyme, EcA2-4M and EcA2-K12, shedding light on the potential implications for their therapeutic applications. The EcA2-4M has been used in therapy since its FDA approval in 1978, while EcA2-K12 is a commonly used variant in the scientific literature.^9^

None of the spectroscopic measurements (circular dichroism, fluorescence), crystallography, or even the high-sensitivity ion mobility was capable of discerning between the two EcA2 variants, which are shown here to have well-defined differences in their catalytic properties. These data evidence the limitations of such state-of-the-art techniques in addressing clear differences in enzyme function, and emphasize the need for exhaustive and irrefutable proof of similarity or differences between follow-on biopharmaceuticals. International regulation should address this limitation, giving proper emphasis to activity, preferably in vivo, and amino acid sequencing in the comparability exercise.

There are few variants of EcA2 in therapeutic use, and their interchangeability has been a focus of scientific literature, particularly due to discrepancies in their pharmacology.^34^ Recently we focused on describing a case involving the substitution of Aginasa with Leuginase.^25,26,35^ The former is identical to EcA2-4M, while the latter, which was considered substandard in terms of therapeutic outcomes, has two amino acid residue mutations compared to EcA2-4M. Interestingly, a similar outcome was described in 1996 with Crasnitin from Bayer, which has the same amino acid sequence as Leuginase, and Asparaginase from Medac, which has the same sequence as Aginasa.^34^ Notably, both l-asparaginases from Leugianse and Aginasa exhibited similar enzymatic activity in vitro, despite the substantially higher in vivo clearance of Leuginase. Despite the differences in their amino acid sequences, the reason for the discrepant pharmacological outcomes remains an open question.

As presented in this manuscript, the EcA2-4M variant exhibits more than twice the specific activity and cytotoxicity of EcA2-K12. This difference in activity is most likely due to the higher substrate-to-product conversion rate exhibited by EcA2-4M, as indicated by the catalytic constant (Table 1 and Figure 2).

**Table 1:** Kinetics of l-Asparaginases^a^

	l-Asn			l-Asp	l-Gln		
	*k*_cat_ (s^–1^)	*K*_M_ (μM)	*k*_cat_/*K*_M_ (μM^–1^ s^–1^)	*K*_D_ (μM)	*k*_cat_ (s^–1^)	*K*_M_ (μM)	*k*_cat_/*K*_M_ (μM^–1^ s^–1^) ×10^–3^
K12	26 ± 2	8 ± 3	4 ± 2	211 ± 52	0.39 ± 0.07	2301 ± 75	0.17 ± 0.04
4M	60 ± 7	11 ± 5	8 ± 7	66 ± 14	3.3 ± 0.5	3111 ± 159	1.1 ± 0.2
fold change	2.3 ± 0.3	1 ± 1	2 ± 2	0.3 ± 0.1	8 ± 2	1.35 ± 0.08	6 ± 2
*p*-value	0.000085	0.404188	0.390079	0.009563	0.000191	0.000092	0.000569

a The values are expressed as mean and the standard deviation of four independently measured experimental data. The following equation was used for the calculation of the fold change standard deviation of the kinetics data: $\mathrm{SD}=R\times \sqrt{{\left(\frac{{\mathrm{SD}}_{A}}{N_{A}}\right)}^{2}+{\left(\frac{{\mathrm{SD}}_{B}}{N_{B}}\right)}^{2}}$ where SD is the standard deviation, *R* is the result of the ratio between two values, SD_A_ and SD_B_ are the standard deviations of each value, and *N*_A_ and *N*_B_ are the values being compared. The *p*-value was calculated for the comparison of the kinetic/thermodynamic constants using the Student’s *t*-test.

EcA2-K12 and EcA2-4M variants exhibit similar apparent affinity (*K*_M_) for l-Asn. Notably, determining such a low *K*_M_ for EcA2 (around tens of micromolar) is challenging due to the need for highly sensitive methods. The most commonly used method involves the discontinuous detection of ammonium by the Nessler reagent, which is described to be troublesome to give reproductive results.^36–38^ In this work, we used 1D [^1^H]-NMR to directly and continuously monitor the conversion of l-Asn to l-Asp and analyzed the data using the Lambert W function, which is a direct solution of the integrated Michaelis–Menten equation.^39–44^

Interestingly, even though the conversion of l-Asn to l-Asp bound to the active site of EcA2-4M, is more efficient, EcA2-K12 has an approximately 3-fold higher *K*_D_ for the product l-Asp than EcA2-4M. This lower binding affinity is accompanied by a less efficient closing of the conformationally flexible active site N-terminal loop (residues 18–31) and hinge region (residues10–17) on the catalytic site of EcA2-K12. The N-terminal loop and hinge region contain amino acid residues directly involved in catalysis (T12 and Y25), making their stabilization in the closed conformation fundamental for the interconversion of l-Asn to l-Asp.^10,45,46^

According to mutational studies on EcA2 activity, besides the two aforementioned residues, T89, D90, and K162 are critical for catalysis, while S58 and D90 are the most significant residues for substrate binding and Q59, N248, and E283 influence substrate binding.^10,29,36,38,46^ None of these residues show significant difference in their positions according to the 3D crystal structures determined here. However, the residues in positions 27 (V or A) and 64 (N or D) exhibit significant differences in their positions relative to the product l-Asp bound to the active site of EcA2. This indicates that these two residue replacements might be the primary factors responsible for the changes in the kinetic parameters of the EcA2 variants studied. Additionally, among the amino acid sequence differences between EcA2-K12 and EcA2-4M, only the amino acid at position 27 is located within the enzyme active site (Val27 in K12 and Ala27 in 4M), more precisely in the N-terminal flexible loop. Our group previously demonstrated that the residue at position 27 is essential for stabilizing the conformational dynamics of the N-terminal loop, which acts as a lid for the catalytic site.^26^ This stabilization could explain the observed differences in the *k*_cat_ of the analyzed variants. We found one report showing that the relative activity of the EcA2-K12-V27A site-directed mutant is the same as that of the wild-type EcA2-K12.^10^ However, the enzymatic assay was performed using the alternative substrate l-aspartic acid β-hydroxamate with unpurified samples, and caution must be taken when extrapolating this result to the substrates l-Asn or l-Gln. Another study showed that the EcA2-K12-V27T mutant has an approximately 4-fold decreased turnover number compared to the wild-type enzyme for l-Gln but did not show any change in the turnover number for l-Asn.^47^ This finding supports the importance of the residue at position 27 for the activity of EcA2. Nevertheless, the crystallographic data in that report showed that the conformations of the mutant and native enzymes bound to l-Asp align very precisely, indicating that the main reason for the influence on the activity is the replacement of one methyl group of Valine by a hydroxyl of Threonine.

The explanation for the distinct activities of EcA2-K12 and EcA2-4M requires further investigation, and this represents a limitation of the study. Additional crystallographic data with nonhydrolyzable or slowly hydrolyzable analogs of l-Asn, which we are not aware of but may include d-Asn,^48^ would be valuable. We also note that incorporating mutants that are unable to convert l-Asn to l-Asp (such as those involving residues T12, Y25, T89, D90, and K162) or using solution conditions that significantly reduce enzyme activity could be useful. However, the use of these options is not without potential risks, including the introduction of artifacts that could lead to misinterpretation of the mechanism underlying the lower *k*_cat_ of EcA2-K12 compared to EcA2-4M.

The *K*_M_ values determined for l-Gln were very similar for both enzyme variants. However, considering the turnover rates, the EcA2-K12 variant is more specific for l-Asn, with a *k*_cat_^Asn^/*k*_cat_^Gln^ ratio of 66.7 ± 0.2 (Table 1). In contrast, the EcA2-4M variant shows lower specificity for l-Asn, with a *k*_cat_^Asn^/*k*_cat_^Gln^ ratio of 18.2 ± 0.2. The much lower catalytic efficiency of EcA2-K12 for l-Gln, which has an extra CH_2_ group in its side chain compared to l-Asn, is likely due to steric hindrance caused by the bulkier side chain of the valine at position 27 compared to the alanine in EcA2-4M, which allows better accommodation of l-Gln. Indeed, crystal structures of EcA2 show that the binding of l-Gln is less efficient because of the misfitting of this amino acid in the tight active site of EcA2.^49^ Therefore, the N-terminal flexible loop is not well-defined in the complex of EcA2 with l-Gln, and this is the main cause for the much lower turnover rate for this amino acid.

The l-Asn/l-Gln specificity ratio is of great importance for l-asparaginase therapy. The l-glutaminase activity, while apparently essential for complete antileukemic efficacy on tumor cells with significant asparagine synthetase expression,^50–52^ is not required for anticancer activity against malignant cell lineages without asparagine synthetase expression.^53^ Nevertheless, the l-glutaminase activity is associated with several significant toxic side effects. These include immunosuppression, hepatotoxicity, encephalopathy, pancreatitis, and coagulation dysfunction, all attributed to the depletion of serum l-Gln and concomitant production of ammonium.^54^ Ammonium ion is one of the products of the hydrolysis of both l-Asn and l-Gln. Since the plasmatic concentration of l-Gln [419–1008 μM^55^ is much higher compared to l-Asn [28–140 μM^55^, l-Gln hydrolysis will have a more profound effect on the eventual rise in ammonia concentration, potentially leading to harmful levels for the patient.^56^ Therefore, engineering or identifying enzymes that retain asparaginase activity while reducing glutaminase activity would be advantageous in minimizing these toxic effects and is indeed a focus of research by many groups.^12,18^ Nevertheless, there is currently no clinical use of engineered asparaginase enzymes with significantly reduced glutaminase activity, which would help avoid deleterious therapeutic side-effects during leukemia treatment. EcA2-K12 is a valuable candidate for this. Due to its 4-fold higher *k*_cat_^Asn^/*k*_cat_^Gln^, EcA2-K12 might offer a better therapeutic window than EcA2-4M. However, the latter enzyme is still a good therapeutic choice and is indeed the most used enzyme in clinical practice, probably due to its higher turnover rate that causes a faster decrease in l-Asn concentration. Nevertheless, caution is necessary with the therapeutic schemes using EcA2-4M since its faster action on l-Asn [normal plasmatic concentrations around 84 μM] can cause a significantly harmful increase in the ammonium plasma concentration [above 50 μM^57^.

No significant alterations were observed in the global conformation and thermal stability of the two enzymes variants, suggesting that the differences in the amino acid sequences of each variant do not result in major interferences to the protein’s 3D structure. The collected 1D [^1^H]-NMR spectra demonstrated that both EcA2 variants are purified with similar, if not identical, open conformations of the enzyme N-terminal catalytic loop, as indicated by the presence of several sharp and well-defined peaks throughout their spectra, originating from signals of residues in this flexible N-terminal loop, particularly the characteristic signals of the aromatic ring of Tyr25 at approximately 7.1 and 6.9 ppm.^29^ Nonetheless, size exclusion chromatography experiments indicated a higher propensity for EcA2-K12 to form oligomers, with prepeaks accounting for 13% of the total peak area compared to 5% for EcA2-4M. This propensity is very similar to what we previously observed using size exclusion chromatography and small-angle X-ray scattering for two EcA2 biopharmaceuticals,^26^ and could significantly impact several enzyme characteristics, including long-term storage, plasmatic activity, immunogenicity, and plasma half-life. In our analysis of activity and binding, we assume 100% homotetrameric assemblies of EcA2 with four active sites. While our data cannot definitively determine which species binds the substrate, part of the differences in activity, including binding to l-Asp, may be attributed to differences in oligomerization between the two EcA2 variants.

## Conclusions

Although our current in vitro cell cytotoxicity assays indicate that the EcA2-4M variant has IC_50_ values twice those found for the EcA2-K12 variant, further animal in vivo and patient ex vivo assays are required prior to further conclusions and go/no-go decision taking for biosimilar developments. While the EcA2-4M has become a reference as biopharmaceutics for acute lymphoblastic leukemia (including Elspar, Aginasa, Spectrila, Oncaspar and Asparlas), the present data demonstrate that other variants from strains, including K12, may represent a source of biosimilars with properties that fulfills specific patient needs.

## Materials and Methods

### Recombinant Production of l-Asparaginase 2 Variants

The cDNA encoding the l-asparaginase type 2 variants (consisting of the protein mature sequence including amino acids 23 to 348) were synthesized with the pelB leader peptide at the N-terminal region, optimized for expression in . The synthesized cDNA was cloned into pET25b plasmid by Genscript. Each construct was transformed into competent BL21­(DE3) cells (Sigma-Aldrich, St. Louis, MO) using standard heat shock protocols. For the expression of EcA2 variants, transformed cells were cultured in Terrific-Broth (TB) medium, with 50 μg/mL ampicillin, and incubated at 37 °C until an optical density at 600 nm of 4.5 was reached. At this point, IPTG was added to a final concentration of 0.2 mM, and cells were incubated for 20 h at 37 °C.

To purify the EcA2 variants, the cells were separated from the medium by centrifugation at 8000*g* for 30 min. The supernatant was filtered sequentially using 0.45 and 0.22 μm membranes. The filtered supernatant was then concentrated 2.5× by ultrafiltration, using a 30 kDa membrane (Pellicon XL Cassette, Merck Millipore, Germany), and a tangential flow filtration system (Pall Minimate, Pall Corporation, USA), with a flow rate of 50 mL/min and a pressure of 1.5–2.0 bar. The supernatant was diafiltrated in the same system with 10 volumes of 25 mM sodium phosphate buffer, pH 7.4.

Ammonium sulfate was added to the sample under slow agitation in an ice bath to a final concentration of 1 M. The sample was then loaded onto a Phenyl Sepharose CL-4B resin at 4 mL/min, pre-equilibrated with 25 mM sodium phosphate buffer, pH 7.4, containing 1 M ammonium sulfate. The unbound material was eluted with the same buffer and then, bound proteins were eluted with a decreasing gradient of ammonium sulfate (1–0.4 M). Fractions containing the proteins of interest, as analyzed by SDS–PAGE, were pooled and diafiltrated against 25 mM sodium phosphate buffer, pH 7.4, until the conductivity reached 4.0 mS/cm, using tangential flow filtration with a 30 kDa Millipore membrane (Pellicon XL Cassette, Merck Millipore, Germany). The sample was then loaded onto a 5 mL HiTrap Q FF anion exchange chromatography column (Cytiva, USA), pre-equilibrated with 25 mM sodium phosphate buffer at pH 7.4. Proteins were eluted from the column with a gradient of 0–300 mM sodium chloride. Fractions containing the proteins of interest, as analyzed by SDS–PAGE, were pooled, concentrated by ultrafiltration, and stored at −20 °C. Protein concentration was determined spectrophotometrically using the molar extinction coefficient of 23,505 M^–1^·cm^–1^ at 280 nm.

### Asparaginase Activity Assay

The activity of l-asparaginase was determined by monitoring the decrease in amide bond absorption at 225 nm using a JASCO V-730 UV–visible spectrophotometer (JASCO, Inc.) with quartz cuvettes of 1 cm optical path length. The assay was conducted in 100 mM sodium phosphate buffer at pH 8 and 25 °C, containing 20 mM of l-Asn. Initially, the hydrolysis of l-Asn in the absence of the enzyme was monitored. Then, 50 μg of l-asparaginase from a solution at 2 mg/mL was added to the cuvette, and the hydrolysis was monitored over time. The absorption coefficient of l-Asn at 225 nm was experimentally obtained by constructing a standard curve using concentrations ranging from 2 to 20 mM in the same buffer and under the same conditions used for the activity assays. One unit (U) of asparaginase activity is defined as the amount of enzyme that catalyzes the hydrolysis of 1 μmol of l-Asn per minute under the assay conditions. Measurements were performed in triplicate for three samples. Standard deviations were calculated from the experimental data using standard mathematical approaches available in GraphPad Prism 8 (GraphPad Software, USA).

### Activity on Lymphoblast Cells

The effect of l-asparaginase type 2 variants was assessed using human lymphoblast cells (CCRF-CEM), obtained from the Rio de Janeiro Cell Bank (BCRJ #0063). The cells were cultured in RPMI-1640 medium with 10% fetal bovine serum. A fixed number of cells (10̂4) were incubated for 48 h at 37 °C in 96-well plates with varying concentrations of each EcA2 variant (0.3, 1.0, 3.0, 10.0, and 30.0 μg/mL). Cytotoxicity was then evaluated using the MTT reduction assay (3-(4,5-dimethylthiazol-2-yl)-2,5-diphenyl tetrazolium bromide, Sigma-Aldrich).^58^ After the treatment period, MTT solution was added to the culture medium to a final concentration of 0.5 mg/mL and incubated in the dark at 37 °C for 4 h. Following incubation, the MTT solution was removed, and the resulting crystals were dissolved in DMSO. Colorimetric analysis was performed using a plate reader (Multiskan Thermo Scientific) at 570 nm. The results were normalized and expressed as percentages relative to the absorbance of untreated cells.

### Kinetics Experiments by 1D ^1^H NMR

To determine the kinetic parameters of the two EcA2 variants, 160 μM of l-Asn or 640 μM of l-Gln was used, in 100 mM sodium phosphate buffer at pH 8.0, 10% D_2_O, and 0.2 mM sodium 3-(trimethylsilyl)-1-propanesulfonate (DSS), in a final volume of 600 μL in a 5 mm NMR tube, at 25 °C. The 1D ^1^H NMR spectra were recorded in a Bruker Ascend 700 MHz spectrometer equipped with a triple-resonance, *Z*-axis gradient 5 mm probe (Bruker Spin Corp., GmbH), using a pulse sequence with excitation sculpting to suppress the water signal, acquiring 64 transients with a recycle delay of 1.5 s. After collecting the spectrum at baseline, the kinetics were initiated by mixing the enzymes to a final concentration of 1.0 nM of the EcA2 4 M variant, or 2.1 nM of the EcA2 K12 variant when using l-Asn, and 1.3 μM of the EcA2 4 M variant, or 2.5 μM of the EcA2 K12 variant when using l-Gln. The spectra were then collected sequentially until the maximum time necessary for each kinetic experiment to reach substrate depletion. The spectra were processed and analyzed using TopSpin 4.0.6 (Bruker Spin Corp., GmbH). The areas of the multiplets corresponding to the Hα of l-Asn and the Hγ of l-Gln were normalized relative to the area of the DSS methyl signal. The concentrations of these amino acids throughout the reactions were calculated from the ratio of the normalized multiplet areas to the maximum added concentration of each amino acid. A nonlinear regression model using the Lambert W eq (eqs 2 and 3) was used to fit the substrate consumption or product formation by the variants^39,59^

1 $$\left[S\right]=K_{M}\;\ln\left[1+\left\{\frac{{\left[S\right]}_{0}}{K_{M}}\;\exp\left(\frac{{\left[S\right]}_{0}-V_{\max}t}{K_{M}}\right)\right\}\right]\times \frac{1-\ln\left[1+\ln\left[1+\left\{\frac{{\left[S\right]}_{0}}{K_{M}}\;\exp\left(\frac{{\left[S\right]}_{0}-V_{\max}t}{K_{M}}\right)\right\}\right]\right]}{2+\ln\left[1+\left\{\frac{{\left[S\right]}_{0}}{K_{M}}\;\exp\left(\frac{{\left[S\right]}_{0}-V_{\max}t}{K_{M}}\right)\right\}\right]}+\mathrm{offset}$$

2 $$\left[P\right]={\left[S\right]}_{0}-\left(K_{M}\;\ln\left[1+\left\{\frac{{\left[S\right]}_{0}}{K_{M}}\;\exp\left(\frac{{\left[S\right]}_{0}-V_{\max}t}{K_{M}}\right)\right\}\right]\times \frac{1-\ln\left[1+\ln\left[1+\left\{\frac{{\left[S\right]}_{0}}{K_{M}}\;\exp\left(\frac{{\left[S\right]}_{0}-V_{\max}t}{K_{M}}\right)\right\}\right]\right]}{2+\ln\left[1+\left\{\frac{{\left[S\right]}_{0}}{K_{M}}\;\exp\left(\frac{{\left[S\right]}_{0}-V_{\max}t}{K_{M}}\right)\right\}\right]}+\mathrm{offset}\right)$$

These equations describe the relationship between substrate ([S]) or product concentration ([P]) and time (*t*) during the reaction, starting from an initial substrate concentration ([S]_0_), and was used to determine the maximum enzymatic velocity (*V*_max_) and the Michaelis–Menten constant (*K*_M_). The Lambert W equation shown above was implemented in GraphPad Prism 8 (GraphPad Software, USA) using the following expressions (eqs 4 and 5)­

3 $$y=K_{m}\times \ln\left(1+S0/K_{m}\times \exp\left(\left(S0-V_{m}\times x\right)/K_{m}\right)\right)\times (1-\ln(1+\ln(1+S0/K_{m}\times \exp(\left(S0-V_{m}\times x\right)/K_{m})))/(2+\ln(1+S0/K_{m}\times \exp(\left(S0-V_{m}\times x\right)/K_{m}))))+\mathrm{offset}$$

4 $$y=S0-(K_{m}\times \ln\left(1+S0/K_{m}\times \exp\left(\left(S0-V_{m}\times x\right)/K_{m}\right)\right)\times (1-\ln(1+\ln(1+S0/K_{m}\times \exp\left(\left(S0-V_{m}\times x\right)/K_{m})))/(2+\ln\left(1+S0/K_{m}\times \exp\left(\left(S0-V_{m}\times x\right)/K_{m}\right)\right)))+offset\right)$$

This equation was fitted to the experimental data using GraphPad Prism 8 (GraphPad Software, USA) to obtain the *V*_max_ values. Subsequently, the *K*_M_ was determined using the same equation, defining the average *V*_max_ values obtained. Four distinct reactions were evaluated for each tested condition.

### Circular Dichroism

Circular dichroism experiments were conducted using a Chirascan CD spectropolarimeter (Applied Photophysics, Leatherhead, UK) with a 100 μm quartz cuvette. Far-UV spectra were recorded from 190 to 260 nm at 25 °C using Eca2 variants at 5 μM diluted in 25 mM sodium phosphate buffer at pH 7.4. Spectral resolution was set at 0.5 nm, scan speed at 100 nm/min and three accumulations were averaged. The final data were obtained by subtracting the buffer signal for background correction and were reported as molar ellipticity per residue (deg·cm^2^·dmol^–1^).^60^ The data were processed using GraphPad Prism 8 (GraphPad Software, USA).

### Protein Fluorescence

Protein fluorescence assays were performed using a Jasco FP-8250 spectrofluorometer (Jasco, Japan) with a 10 mm quartz cuvette. Fluorescence spectra were obtained using excitation set at 295 nm and emission recorded from 300 to 400 nm using 20 μM of each EcA2 variant diluted in 25 mM sodium phosphate buffer at pH 7.4. The spectral resolution was set to 0.5 nm, scan speed to 100 nm/min, and two accumulations were averaged. Thermal denaturation curves were monitored by fluorescence emission at 340 nm as the sample was heated from 25 to 80 °C in 0.5 °C increments, using a temperature change rate of 1 °C/min.

Data were fit to a two-state model equation with baseline correction (eq 6)^61,62^

5 $$F=\frac{\left(F_{N}+S_{N}T\right)+\left(F_{U}+S_{U}T\right)\times e^{\left[-\left(\Delta H_{m}\left(1-{T/T}_{m}\right)\right)\right]/\left(RT\right)}}{1+e^{\left[-\left(\Delta H_{m}\left(1-{T/T}_{m}\right)\right)\right]/\left(RT\right)}}$$

This equation describes the relationship between observed fluorescence signal (*F*) and temperature (*T*) in kelvin, and it was used to determine the melting temperature (*T*_m_) of the EcA2 samples. *F*_N_ and *F*_U_ are the protein fluorescence signals in the native and unfolded states, respectively, which were numerically assigned values of 0 and 1 as the experimental data were normalized. *S*_N_ and *S*_U_ denote the calculated slopes of the pre- and postdenaturational phases, respectively. Δ*H*_m_ is the calculated enthalpy of denaturation at the transition midpoint, and *R* is the ideal gas constant (1.987 cal·mol^–1^·K^–1^). This equation was implemented in GraphPad Prism 8 (GraphPad Software, USA) using the following expression (eq 7)­

6 $$Y=\left(\left(F_{N}+\left(S_{N}\times \left(X+273.15\right)\right)\right)+\left(\left(F_{U}+\left(S_{U}\times \left(X+273.15\right)\right)\right)\times \exp\left(-\frac{\left(H_{m}\times \left(1-\left(\frac{\left(X+273.15\right)}{\left(T_{M}\right)}\right)\right)\right)}{\left(R\times \left(X+273.15\right)\right)}\right)\right)\right)/\left(1+\exp\left(-\frac{\left(F_{U}\times \left(1-\left(\frac{\left(X+273.15\right)}{\left(T_{M}\right)}\right)\right)\right)}{\left(R\times \left(X+273.15\right)\right)}\right)\right)$$

To evaluate the affinity of the EcA2 variants for the ligand by intrinsic fluorescence, different concentrations of l-Asp (0, 62.5, 125, 187.5, 250, 312.5, 375, 437.5, 500, 625, 750, 875, 1000, 1964, 2910, 3847, 4776, and 5696 μM) and 20 μM of the variants were used in 100 mM sodium phosphate buffer at pH 8.0 ^37^. The spectral center of mass of each spectrum was obtained using SpectraGryph software Version 1.2.16.1 (available at: http://www.effemm2.de/spectragryph/). The variation of the spectral center of mass of EcA2 with each l-Asp concentration, in comparison to the sample without ligand, was used to plot the binding curves using GraphPad Prism 8 software (GraphPad Software, USA). The experimental data were fit to a Hill eq (eq 8)­

7 $$y=\frac{B_{\max}X^{h}}{{K_{d}}^{h}+X^{h}}$$

Where *y* is the variation in the center of spectral mass of fluorescence, *B*_max_ is the maximum binding capacity, *X* is concentration of l-Asp in μM, *K*_d_ is the dissociation constant and *h* is the Hill slope.

### Protein Conformation and Ligand Binding Analysis by 1D ^1^H NMR

1D ^1^H NMR spectra of EcA2 variants were collected at 25 °C on a Bruker AVANCE 3600 MHz spectrometer equipped with a triple-resonance, *Z*-axis gradient 5 mm probe (Bruker Spin Corp., GmbH). ^1^H chemical shifts were referenced to sodium 3-(trimethylsilyl)-1-propanesulfonate (DSS). NMR spectra were processed and analyzed using TopSpin 4.0.6 software (Bruker Spin Corp., GmbH). NMR samples used for the analysis of l-Asp titration were prepared with 160 μM of each variant in 25 mM sodium phosphate buffer at pH 7.4, with the addition of l-Asp, for acquisition of 1D ^1^H NMR spectra. The STD-NMR samples were diluted in D_2_O to 10 μM for each EcA2 variant, with a 100-fold excess of ligand (1 mM l-Asp). We used the standard parameter setup from Bruker (STDDIFFGP.3), which employs the “stddiffgp19.3” pulse sequence, with a 25 ms spin-lock filter, and water suppression using the 3–9–19 pulse sequence with gradients. Water suppression pulse sequence was used to remove the signal from the residual H_2_O coming from the stock solutions. The on-resonance spectrum was obtained with saturation of the protein signal at approximately 0.87 ppm, corresponding to a strong methyl signal that is far from any signal of the ligand. For the off-resonance spectrum, saturation was performed at −10 ppm, where no detectable protein or ligand signal was observed. A saturation time of 2.5 s was used as previously determined,^26^ with a recycling delay of 4.0 s. Relative STD percentages were obtained with the highest A_STD_ intensity set to 100% and all other STD signals calculated relative to this.

### Analytical Size Exclusion Chromatography

Analytical size exclusion chromatography was performed to determine the hydrodynamic radius of EcA2 variants, using a TSKgel G2000SWXL column (300 × 4.6 mm—Supelco). The samples contained 50 μM of the recombinant enzymes diluted in 25 mM sodium phosphate buffer at pH 7.4 and 100 mM sodium chloride. Proteins were separated at a flow rate of 0.35 mL/min and monitored by OD at 280 nm. The following globular proteins were used to establish a standard curve for elution profile versus hydrodynamic radius: thyroglobulin at 86 Å (Sigma-Aldrich, Cat #T9145), bovine serum albumin–dimer at 45.6 Å and monomer at 36.2 Å (Sigma-Aldrich, Cat #A7906), ovalbumin at 30.5 Å (Sigma-Aldrich, Cat #A5503) and carbonic anhydrase at 21 Å (Sigma-Aldrich, Cat #C4396).

### Direct Infusion ESI–Traveling Wave0-Ion Mobility Spectrometry–Mass Spectrometry (ESI–TWIM–MS)

The asparaginase samples were diluted to 50 μM in 0.1% formic acid in water/acetonitrile (50:50). ESI-TWIM-MS measurements were performed in a Traveling Wave Ion Mobility Mass Spectrometer (TWIM-MS, Synapt G1 HDMS, Waters, UK). Measurements were performed by direct infusion at 20 μL/min or 10 min, scan time 3 s, interscan time 0.02 s, in positive mode with a capillary voltage of 3.0 kV, sampling cone 40, extraction cone 4.0, collision energy 6.0 (trap) and 4.0 (transfer), N_2_ used as mobility gas at 0.4 bar, source temperature 80 °C and gas flow 5.0 mL/min, IMS gas flow 40.0 mL/min, desolvation temperature of 250 °C and flow of 500 L/h. All other data are available upon request. Data collection was performed in the range of *m*/*z* 500–3000 for 5 min. The mass spectrometer calibration was performed with 0.1% phosphoric acid (v/v) in acetonitrile/H_2_O (1:1). Other typical instrumental settings followed a previous study.^63^ Data were analyzed with DriftScope (Waters Corporation, UK).^64^ All measurements were performed at the Laboratory of Proteomics and Mass Spectrometry (UEMP-IBqM-UFRJ), Rio de Janeiro, RJ Brazil.

### Protein Crystallography

Crystals were obtained by sitting-drop vapor diffusion using 1 μL protein solution with 1 μL precipitant solution equilibrated against 80 μL precipitant solution per well in a Corning 3225 multiwell plate using the sparse PEG-ion screening kits (Hampton Research, USA). Protein solution consisted of 7 mg/mL asparaginase II, either K12 or 4M, in the presence of 10 μM l-Asp. Diffraction-quality crystals were obtained within 2 days for both EcA2-K12 and EcA2-4M in 200 mM ammonium acetate, 20% PEG 3350 (Hampton Research PEG Ion I solution 30, “PEG ion I 30”).

The preliminary characterization of the crystals was performed using CuKα radiation in a Photon II detector mounted in a D8-Venture diffractometer (Bruker AXS, Germany) at CENABIO/UFRJ.

Crystals were harvested from drops using 10 μm nylon Spine Loops (MiTiGen) and flash-frozen in liquid nitrogen, and then transferred to a N_2(g)_ stream at 100 K and a flow rate of 10 L/h (Cryostream 700, Oxford Cryogenics, UK). Data collection was performed in the Manacá beamline from the Sirius Brazilian Synchrotron Light Laboratory (LNLS) at Brazil’s National Center for Research in Energy and Materials (CNPEM), Campinas, Brazil, using 1.459 Å (8.4991 keV) radiation. Diffraction data sets were collected on Pilatus 6 M detector (Dectris) with 0.1°/image.

The images were indexed, processed, and integrated with XDS by the autoProc pipeline.^65^ The molecular replacement was performed with 3ECA.pdb (for the K12 data set) and 1NNS.pdb (for the 4 M data set) using MolRep (CCP4). The solved structures were further refined in real-space with C.O.O.T.^66^ and in reciprocal-space with Refmac.^64^ Water molecules were added using C.O.O.T. The structural validation of the final models showed that almost all dihedral angles are within the favored range. The figures were generated with PyMOL (PyMOL Molecular Graphics System, Version 2.0, Schrödinger LLC).

A summary of the crystallographic parameters from data collection and refinement statistics are shown in Table S1. The resulting models were deposited under pdb codes 9DAF (EcA2-K12, crystallized with l-Asp) and 9DAH (EcA2-4M, crystallized with l-Asp). The raw data sets were deposited at https://www.proteindiffraction.org/under the same PDB ID. Analysis of the B-factor was conducted after normalization as previously described.^67^

### Normal Mode Analysis

Conformational normal-mode analysis was performed with the online tool Webnm@ available at https://apps.cbu.uib.no/webnma3.^68^ The trajectory values calculated after obtaining the movement vectors were extracted from the files generated by the server and plotted in GraphPad Prism v. 8.0.2 for Windows (GraphPad Software, San Diego, California USA, www.graphpad.com).

## Supplementary Material



## Data Availability

The crystallographic structure factors and pdb models presented in this paper have been deposited in the Protein Data Bank (PDB) and the raw data in the Integrated Resource for Reproducibility in Macromolecular Crystallography (https://www.proteindiffraction.org/) with the following codes: 9DAF and 9DAH. All other data sets generated during and/or analyzed during the current study are available from the corresponding author on reasonable request.

## Notes

Extended and raw data on all analyses reported in the main figures (PDF)
