# Supplementary material for: Biochemical and Biophysical Divergences between Two l‑Asparaginase II Variants: Potential for Using EcA2-K12 as a Biosimilar
Source: Biochemistry. 2025 Apr 16;64(14):3015–29. doi: 10.1021/acs.biochem.4c00663 (PMC12273708; doi:10.1021/acs.biochem.4c00663)
Supplement: Supplementary file 1 [file bi4c00663_si_001.pdf]

Supporting Information

Biochemical and Biophysical Divergences Between Two *Escherichia coli* l-Asparaginase II Variants: Potential for using EcA2-K12 as a Biosimilar

Talita Stelling de Araujo[1,‡], Anna Catharinna da Costa[1,3‡], Camila Dias Leite da Silva[1], Fernando de Sá Ribeiro[2, 3], Rafael Alves de Andrade[1], Heitor Affonso Paula Neto[4,5], Renato Sampaio Carvalho[4,5], Luís Maurício T. R. Lima[2,3,5,], Marcius da Silva Almeida[1,3]**

[1] Protein Advanced Biochemistry (PAB), Institute of Medical Biochemistry (IBqM) -National Center for Structural Biology and Bioimaging (CENABIO), Federal University of Rio de Janeiro, Rio de Janeiro, RJ, 21941-902, Brazil

[2] Laboratório de Biotecnologia Farmacêutica (pbiotech), Faculdade de Farmácia, Universidade Federal do Rio de Janeiro, Rio de Janeiro, RJ 21941-902, Brazil

[3] Programa de Pós-Graduação em Química Biológica, Universidade Federal do Rio de Janeiro, Rio de Janeiro, RJ, 21941-902, Brazil.

[4] Laboratório de Alvos Moleculares (LAM), Faculdade de Farmácia, Universidade Federal do Rio de Janeiro, Rio de Janeiro, RJ 21941-902, Brazil

[5] Programa de Pós-Graduação em Ciências Farmacêuticas, Faculdade de Farmácia, Universidade Federal do Rio de Janeiro, Rio de Janeiro, RJ, 21941-902, Brazil.

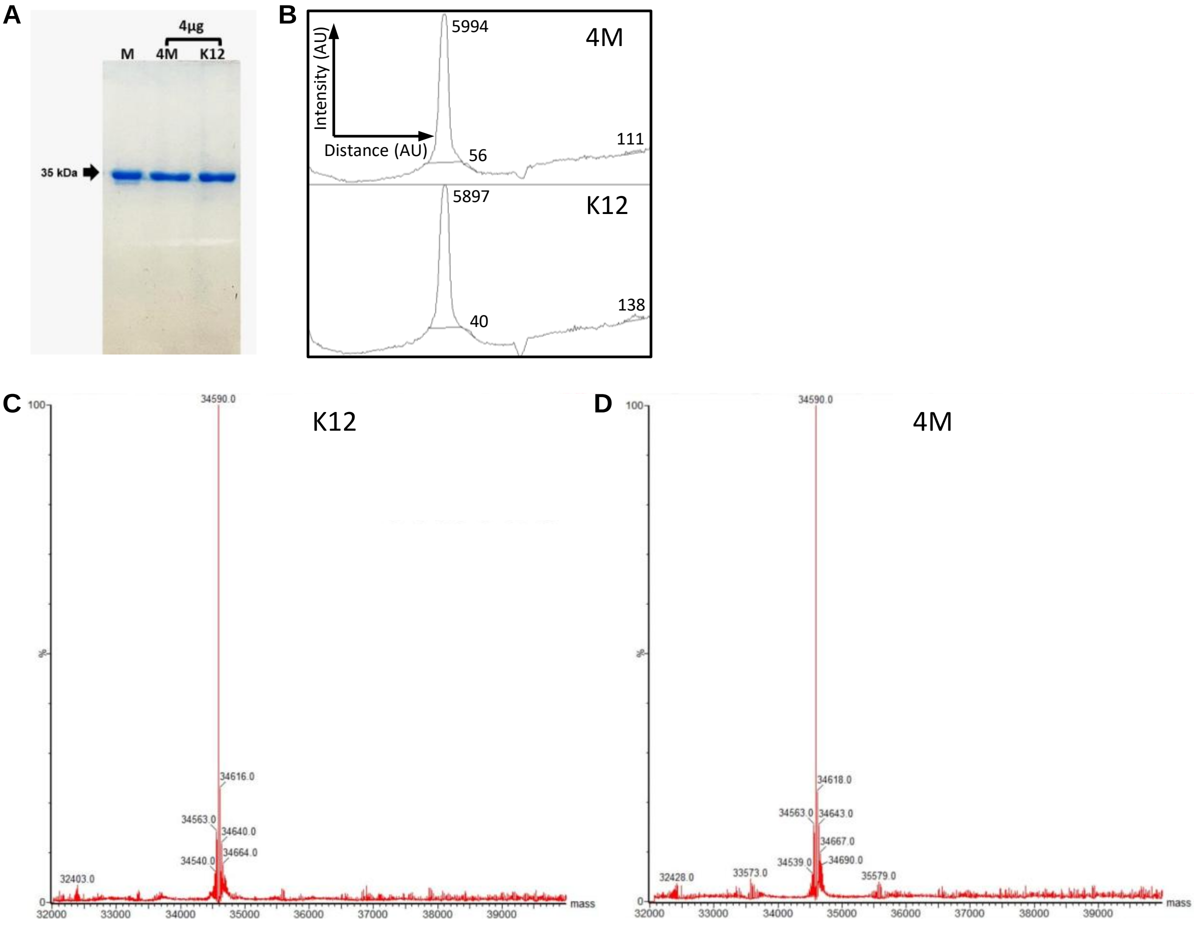

Fig S1. Purity and identity confirmation of purified EcA2. (A) Denaturing 15% polyacrylamide gel electrophoresis with each purified sample (lanes 4M and K12) and the biopharmaceutical Aginasa® as the standard (lane M). (B) Density histograms of the purified sample lanes with purified samples, generated using Image J. The numbers indicate the areas under each peak, with the manually drawn baseline shown as a straight line. (C and D) The EcA2-K12 and EcA2-4M were subjected to direct infusion ESI-MS for determination of intact mass. The deconvoluted spectra are shown.

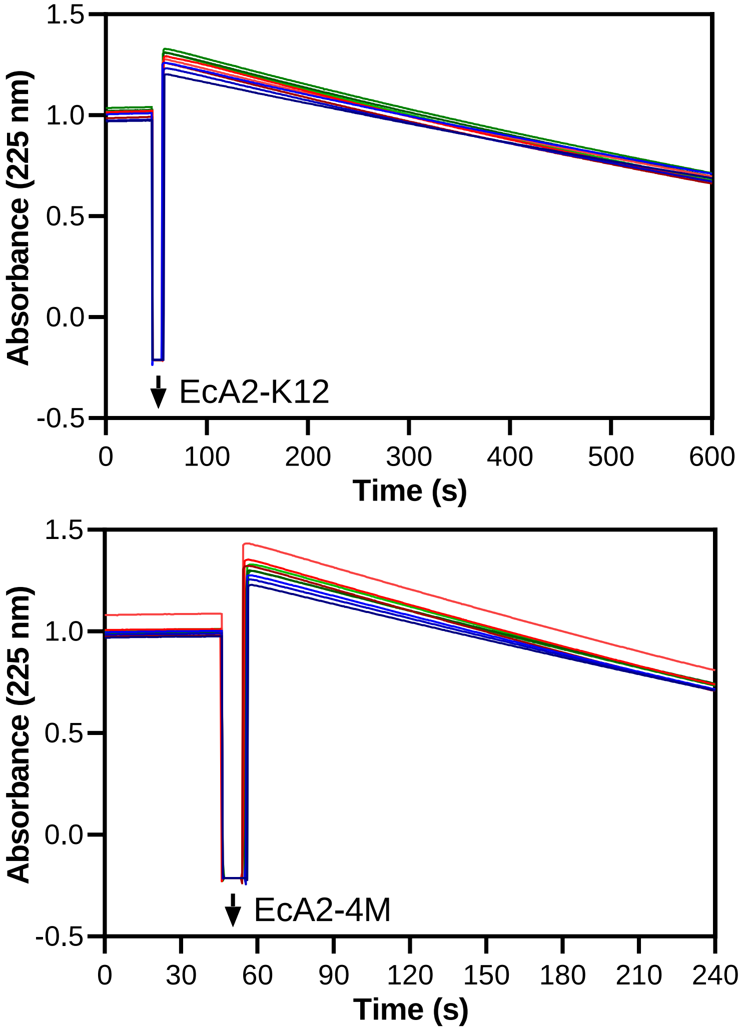

Fig. S2: Spectrophotometric monitoring of L-Asparagine hydrolysis at 225 nm. Absorbance was recorded every 0.5 seconds, with triplicate measurements performed for three independent samples of each EcA2 variant. The addition of each variant is indicated by arrows and labels.

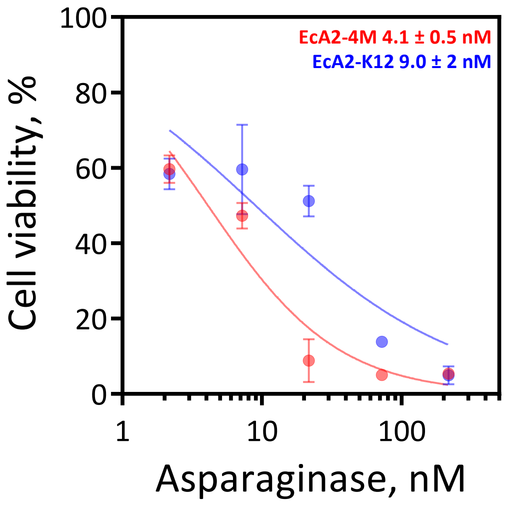

Fig. S3: Cytotoxicity of L-asparaginases. Cytotoxicity was evaluated using the MTT reduction assay and the results were normalized and expressed as percentages of cell viability relative to the absorbance of untreated cells. The experimental data were fit to a dose response equation with variable slope (n = 3, R^2 = 0.92 and 0.97 for EcA2-K12 and EcA2-4M, respectively). Circles represent the mean, and the error bars indicate the standard deviations.

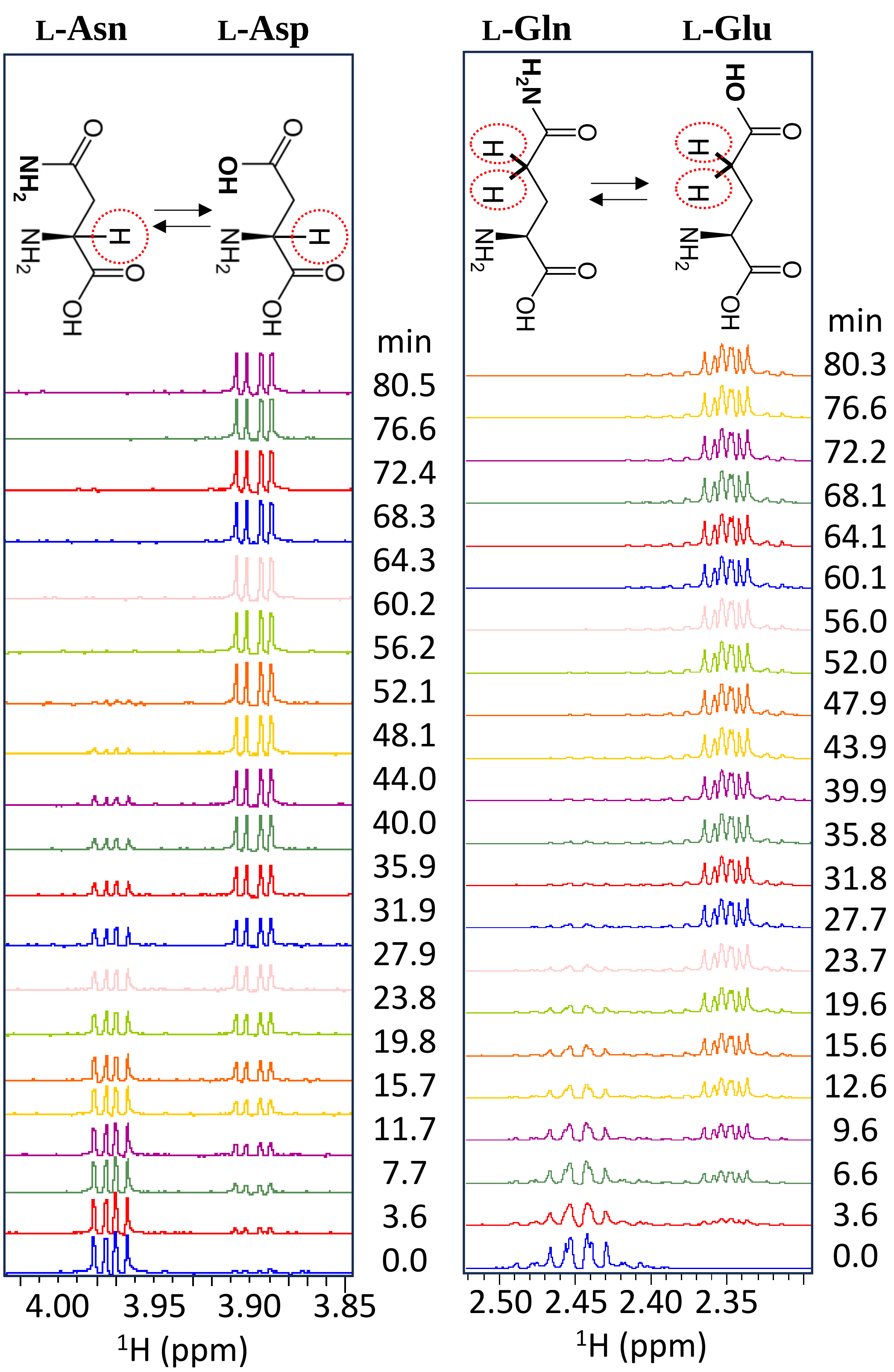

Fig S4. Hydrolysis reaction of L-asparagine or L-glutamine catalyzed by EcA2 variants. monitored by 1D (^{1}H)-NMR. The spectra series over time show a decrease in the intensity of the Hα peaks of L-asparagine and the Hγ peaks of L-glutamine, with a concomitant increase in the Hα peaks of L-aspartate and the Hγ peaks of L-glutamate.

Table S1. Enzymatic kinetics data for the hydrolysis of L-asparagine or L-glutamine and the formation of L-aspartate or L-glutamate catalyzed by EcA2 variants, as monitored by 1D ^{1}H-NMR.

Sample	Parameter	Values				Mean	SD	%D	Fold change (4M/K12)	SD fold change
4M Asn	V_{max} (µM/min)	3.2	3.5	4.2	3.5	3.6	0.4	11	1.1	0.2
	k_{cat} (s^{-1})	54	58	70	58	60	7	11	2.3	0.3
	K_M (µM)	16	14	4	9	11	5	50	1	1
	k_{cat}/K_M (s^{-1} µM^{-1})	3	4	19	6	8	7	88	2	2
4M Asp	V_{max} (µM/min)	3.2	3.0	3.6	3.2	3.3	0.3	8	1.1	0.1
	k_{cat} (s^{-1})	53	50	60	53	54	4	8	2.3	0.2
	K_M (µM)	6.2	5.4	0.2	3.1	3.7	2.7	72	2	2
	k_{cat}/K_M (s^{-1} µM^{-1})	9	9	265	17	75	127	169	2	4
4M Gln	V_{max} (µM/min)	300	207	255	265	257	38	15	4	1
	k_{cat} (s^{-1})	3.9	2.7	3.3	3.4	3.3	0.5	15	8	2
	K_M (µM)	3216	3254	3068	2904	3111	159	5	1.35	0.08
	k_{cat}/K_M (s^{-1} µM^{-1})	0.0012	0.0008	0.0011	0.0012	0.0011	0.0002	16	6	2
4M Glu	V_{max} (µM/min)	277	168	239	156	210	58	28	5	2
	k_{cat} (s^{-1})	3.6	2.2	3.1	2.0	2.7	0.7	28	10	3
	K_M (µM)	2491	2636	2421	2393	2485	109	4	1.6	0.1
	k_{cat}/K_M (s^{-1} µM^{-1})	0.0014	0.0008	0.0013	0.0008	0.0011	0.0003	28	6	2
K12 Asn	V_{max} (µM/min)	3.1	3.6	3.3	2.9	3.2	0.3	9		
	k_{cat} (s^{-1})	24	29	26	23	26	2	9		
	K_M (µM)	8	1*	5	11	8	3	42		
	k_{cat}/K_M (s^{-1} µM^{-1})	3	ND	6	2	4	2	51		
K12 Asp	V_{max} (µM/min)	3.0	2.9	3.1	2.7	2.9	0.1	5		
	k_{cat} (s^{-1})	24	23	25	22	23	1	5		
	K_M (µM)	1.1	1.7	0.2	5.8	2.2	2.5	113		
	k_{cat}/K_M (s^{-1} µM^{-1})	22	14	108	4	37	48	130		
K12 Gln	V_{max} (µM/min)	50	70	56	610*	59	10	17		
	k_{cat} (s^{-1})	0.33	0.47	0.37	ND	0.39	0.07	17		
	K_M (µM)	2388	2218	2265	2335	2301	75	3		
	k_{cat}/K_M (s^{-1} µM^{-1})	0.00014	0.00021	0.00017	ND	0.00017	0.00004	21		
K12 Glu	V_{max} (µM/min)	37	39	47	38	40	4	11		
	k_{cat} (s^{-1})	0.25	0.26	0.31	0.25	0.27	0.03	11		
	K_M (µM)	1600	1464	1421	1605	1522	94	6		
	k_{cat}/K_M (s^{-1} µM^{-1})	0.00016	0.00018	0.00022	0.00016	0.00018	0.00003	17		

* Outlier excluded from the analysis. (%D) percentage of standard deviation (SD) in relation to the mean. V_{max} and K_M were obtained from the experimental data fit to Lambert W equation. k_{cat} was obtained with the equation k_{cat} = V_{max} / [EcA2]. ND – not determined. The following equation (eq 1) was used for the calculation of the fold change standard deviation of the kinetics data:

$$SD = R \times \sqrt{\left(\frac{SD_A}{N_A}\right)^2 + \left(\frac{SD_B}{N_B}\right)^2}, \quad (1)$$

where SD is the standard deviation, R is the result of the ratio between two values, SD_A and SD_B are the standard deviations of each value, and N_A and N_B are the values being compared.

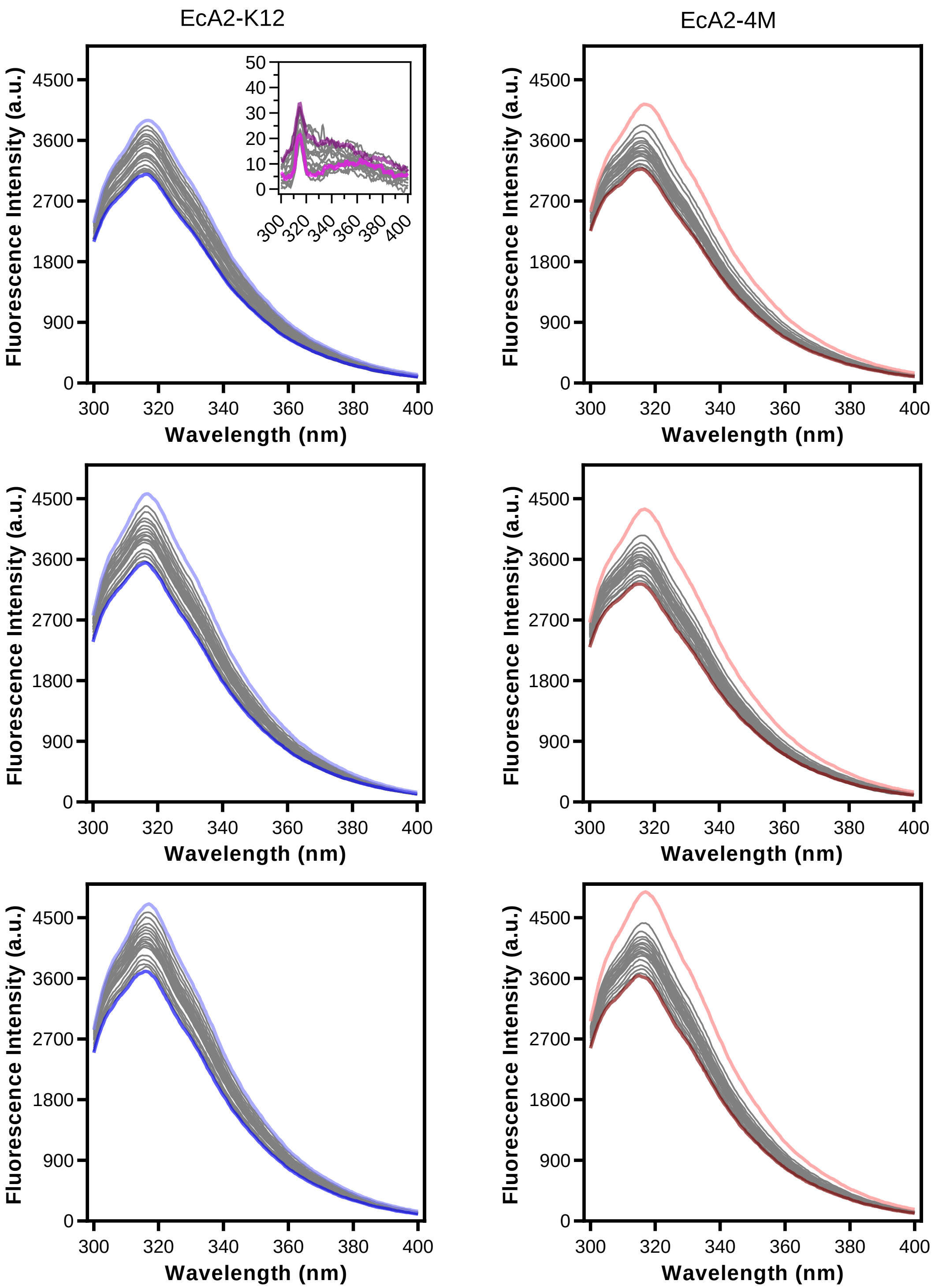

Fig. S5: Overlay of the intrinsic fluorescence spectra of EcA2 variants in the presence of varying L-Asp concentrations. The spectra suffer a blue shift related to the increase of L-Asp concentration (62.5-5,696 µM). Spectra collected without L-asp are shown in light blue or light red, while those collected with the highest L-asp concentration are in dark blue or dark red. Each panel represents the data of one experimental replicate (three for each EcA2 variant). Inset: fluorescence spectra of the buffer at different L-Asp concentration from 0 µM (light purple) to 5,696 µM (dark purple).

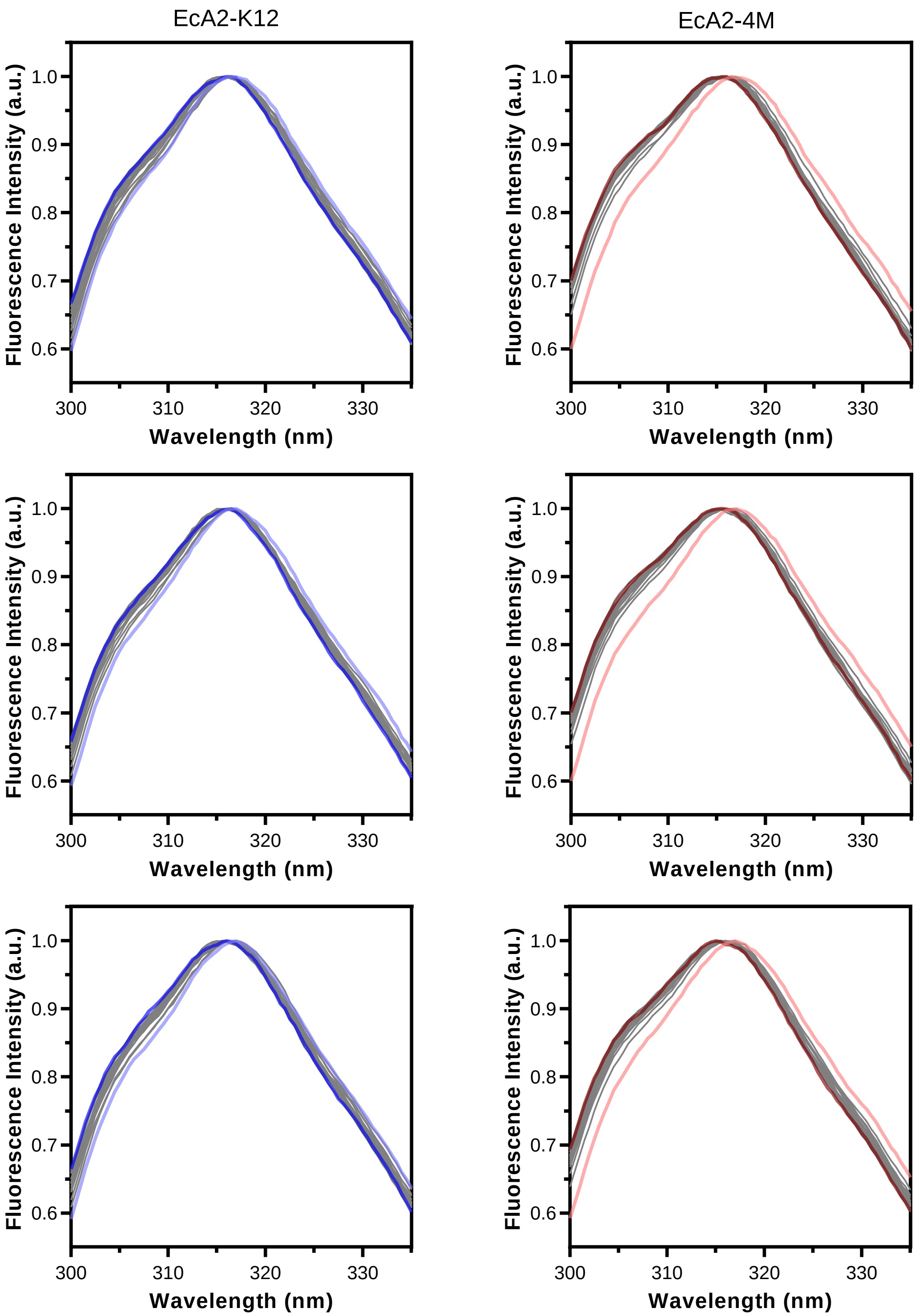

Fig. S6: Overlay of the intensity-normalized intrinsic fluorescence spectra of EcA2 variants in the presence of varying L-Asp concentrations. The spectra suffer a blue shift related to the increase of L-Asp concentration (62.5-5,696 µM). Spectra collected without L-asp are shown in light blue or light red, while those collected with the highest L-asp concentration are in dark blue or dark red. Each panel represents the data of one experimental replicate (three for each EcA2 variant).

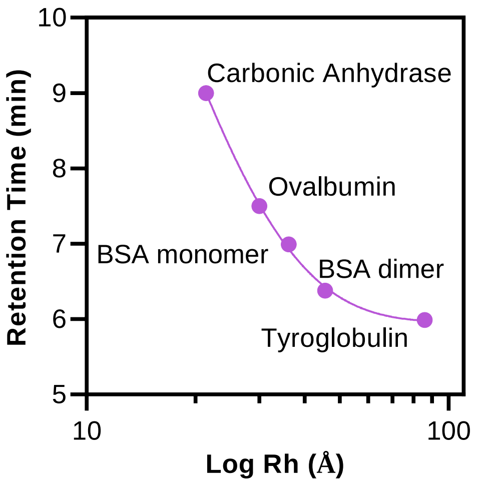

Fig S7. Standard curve of proteins with known hydrodynamic radii run on an analytical size-exclusion chromatography column. Samples were run on a TSKgel G2000SWXL column (300 × 4.6 mm - Supelco).

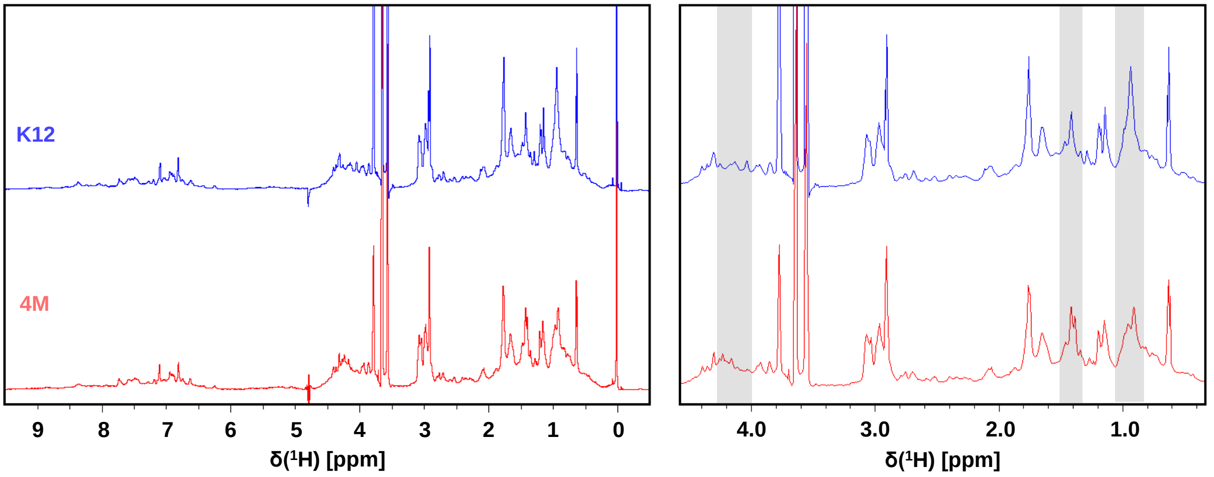

Fig S8. 1D [^{1}H]-NMR spectra of EcA2-K12 (blue) and EcA2-4M (red). NMR spectra were collected at 25 ^{o}C and 900 MHz. Differences among these spectra are highlighted in grey. Signals from DSS standard and glycerol are at 0 ppm and 3.5-3.8 ppm, respectively. Residual signal from water is at 4.7 ppm.

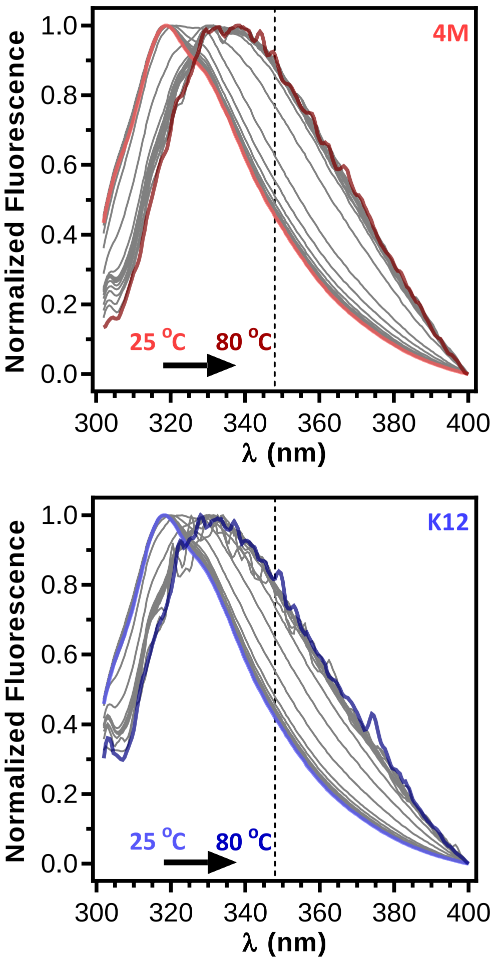

Fig. S9: Thermal denaturation of EcA2 variants monitored by intrinsic fluorescence. The spectral center of mass suffer a red shift related to the increase of temperature (25-80 ºC). Dashed line indicates the intensities at 348 nm, used to calculate the unfolded fraction of protein (shown in Figure 6D).

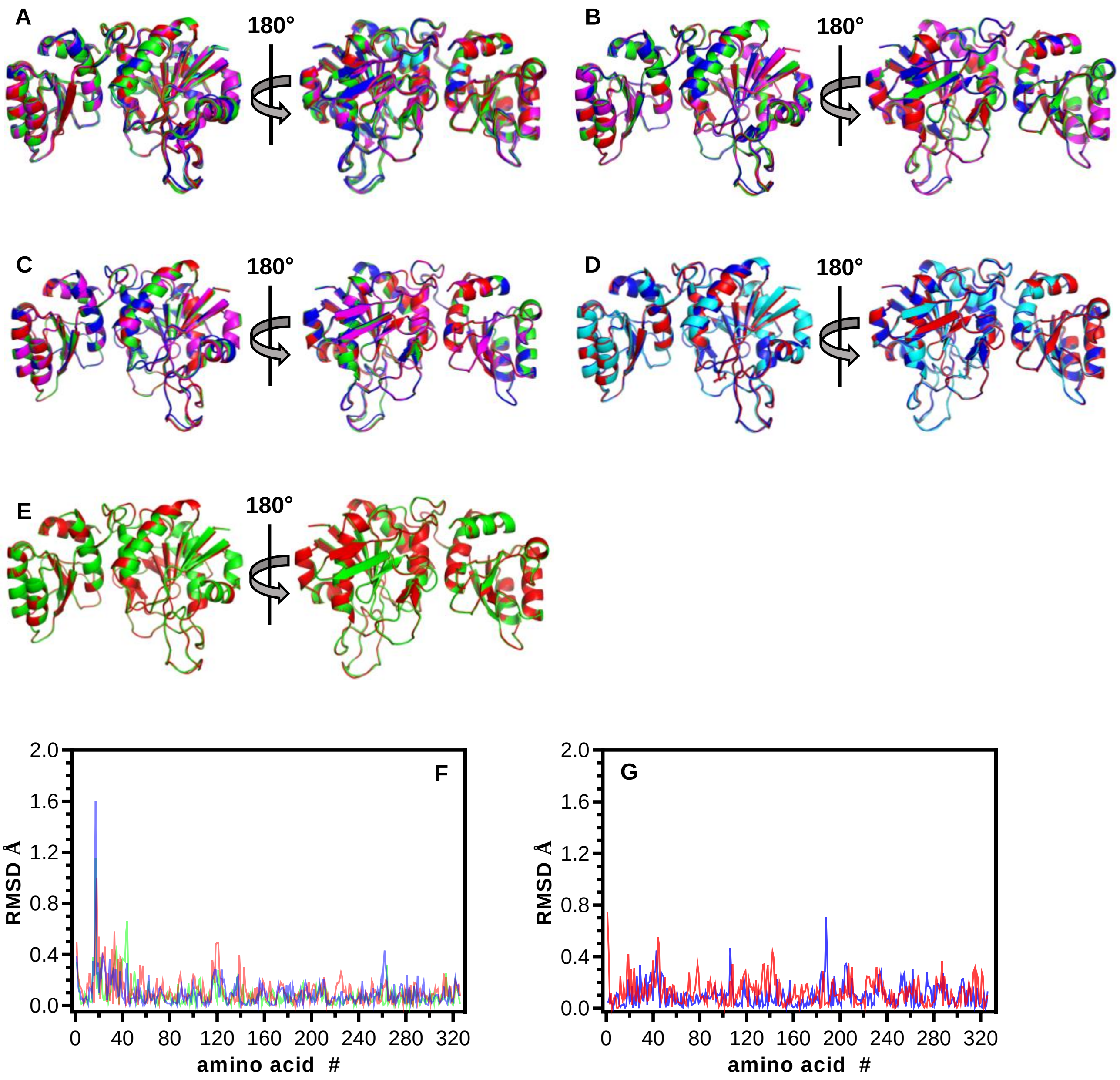

Fig S10. Alignment of L-Asparaginase chains. Structural alignment performed for (A) All chains, 3ECA, EcA2-4M and EcA2-K12. (B) EcA2-K12 chains (C) 3ECA chains. (D) A chains of 3ECA, EcA2-4M and EcA2-K12. (E) EcA2-4M chains. C^{α} RMSD of chain alignment by amino acid residue number of (F) 3ECA chains, A *vs* B (blue); A *vs* C (red); A *vs* D (green), or (G) 3ECA chain A vs EcA2-4M chain A (red); 3ECA chain A vs EcA2-K12 chain A (blue).

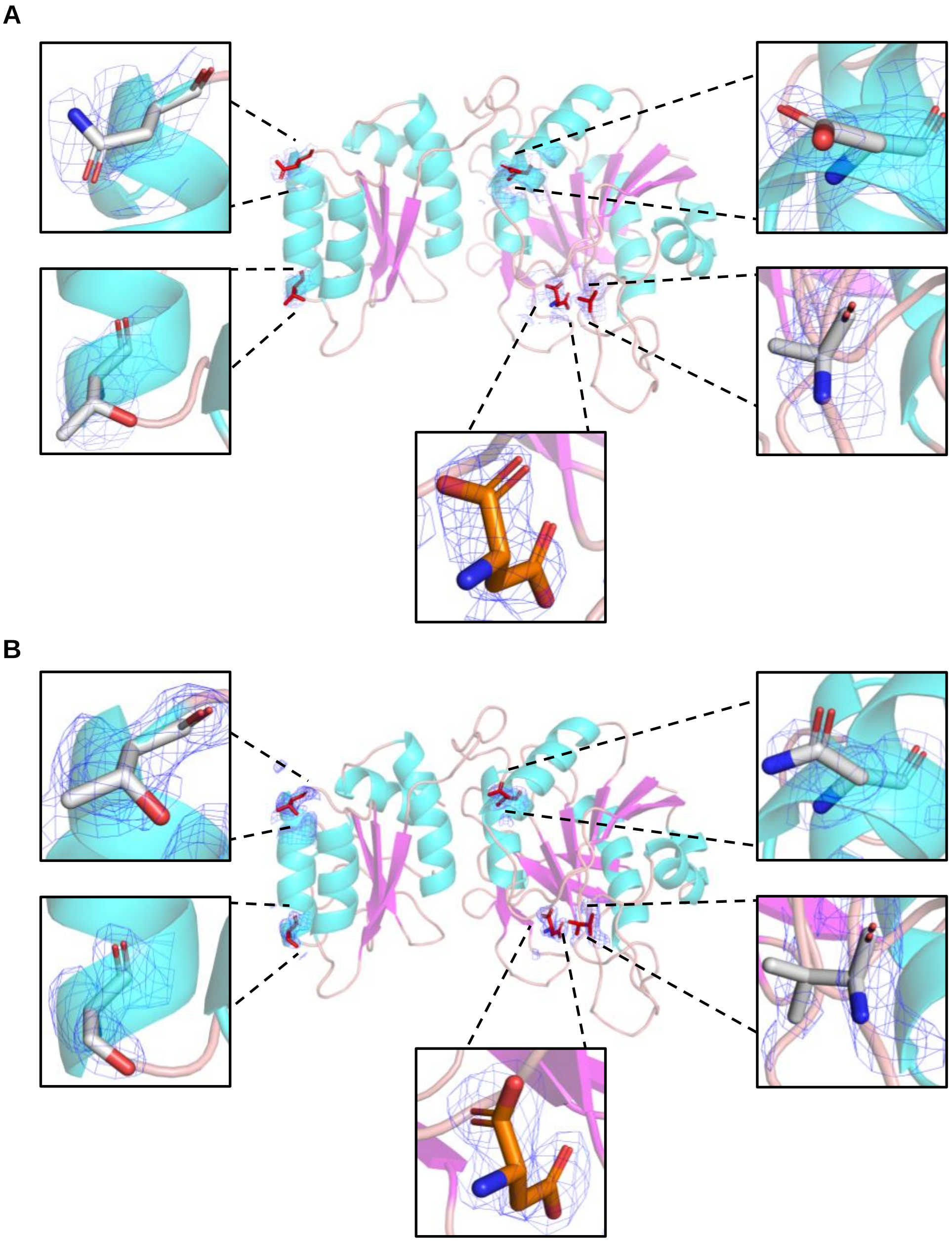

Fig S11. Crystal structures of EcA2-4M and EcA2-K12. Highlighted with the electron density map are the mutated residues and the ligand. (A) EcA2-4M, from left to right and top to bottom, highlight respectively, asparagine, aspartic acid, threonine and alanine (Carbon, nitrogen and oxygen are colored gray, blue and red respectively); Aspartic acid highlighted with the elements carbon, nitrogen and oxygen colored in orange, blue and red respectively. (B) EcA2-K12, from left to right and top to bottom, highlight respectively, threonine, asparagine, serine and valine (Carbon, nitrogen and oxygen are colored gray, blue and red respectively); Aspartic acid highlighted with the elements carbon, nitrogen and oxygen colored in orange, blue and red respectively.

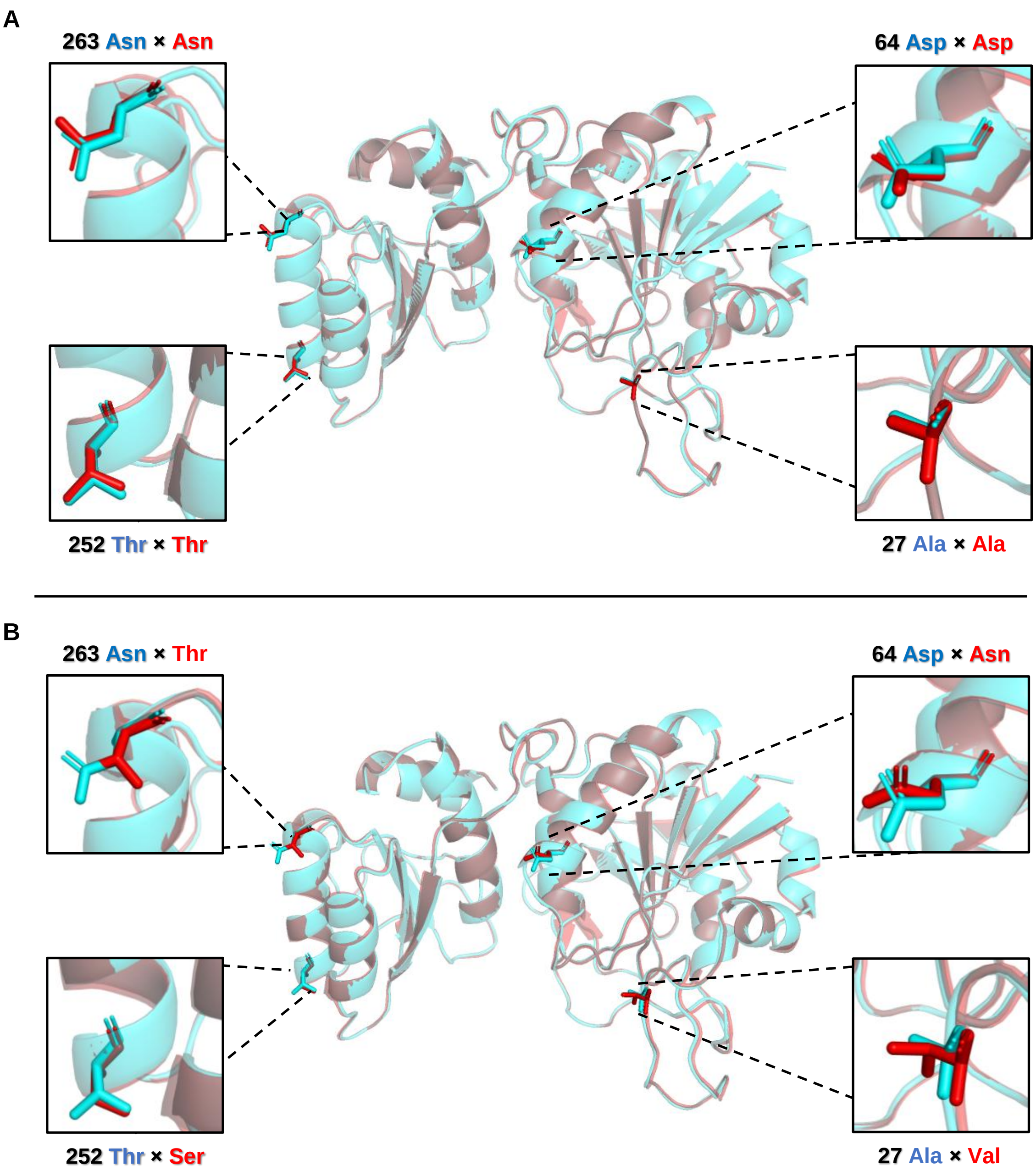

Fig S12. Alignment of L-asparaginase structures highlighting mutated residues. **A)** 3ECA (cyan) *vs* EcA2-4M (red). **B)** 3ECA (cyan) *vs* EcA2-K12 (red).
